# Reactions of nitroxides XIII: Synthesis of the Morita–Baylis–Hillman adducts bearing a nitroxyl moiety using 4-acryloyloxy-2,2,6,6-tetramethylpiperidine-1-oxyl as a starting compound, and DABCO and quinuclidine as catalysts

**DOI:** 10.3762/bjoc.8.171

**Published:** 2012-09-12

**Authors:** Jerzy Zakrzewski

**Affiliations:** 1 Institute of Industrial Organic Chemistry, Annopol 6, 03-236 Warsaw, Poland

**Keywords:** 4-acryloyloxy-2,2,6,6-tetramethylpiperidine-1-oxyl, DABCO, Morita–Baylis–Hillman reaction, nitroxides, quinuclidine

## Abstract

The Morita–Baylis–Hillman adducts bearing a nitroxyl moiety were synthesized from 4-acryloyloxy-2,2,6,6-tetramethylpiperidine-1-oxyl and aliphatic, aryl and heterocyclic aldehydes.

## Introduction

In the Morita–Baylis–Hillman (MBH) reaction, aldehydes react with a double bond activated by an electron-withdrawing group (EWG). The vinylic carbon bearing an EWG undergoes substitution. The reaction is carried out in the presence of either a tertiary amine (e.g., DABCO [2–6], quinuclidine and its derivatives [7–12], DBU [13–14], DBN [13], DMAP and its derivatives [4,15–16], urotropine [17], brucine *N*-oxide [18]) or a phosphine [19] as a catalyst. The MBH reaction is a carbon–carbon bond forming process. This is the reason why a huge amount of research devoted to the reaction is reported every year. The application and scope of the reaction has been summarized in many review articles, e.g., [20], as well as the latest ones [21–24]. Some recent results concerning MBH reaction have been presented [6,16,18,25–33]. MBH adducts themselves are reported to be antiproliferative agents [34]; however, they are often applied as a tool for building more complex target structures, usually of biological importance [35–43]. The MBH reaction is a rather slow process (complete reaction can take hundreds of hours), especially when acrylates are used [44]. As has been very well known since the 1960s, stable nitroxides can react without affecting the unpaired electron. However, there are also reactions that do involve the free electron (e.g., many types of reductions, or a disproportionation in an acidic environment). To the best of our knowledge, nitroxyl radicals have not yet been applied in the MBH reaction (to date), and the potential influence of the nitroxide moiety on the MBH reaction is unknown. Herein we present the MBH reaction with a nitroxyl radical, 4-acryloyloxy-2,2,6,6-tetramethylpiperidine-1-oxyl, used as a starting material as an olefin activated with EWG. Acrylates are considered as rather unreactive in the MBH reaction [44]. It was shown that the effects on the reaction of aryl, benzyl, alkyl, and functionalized alkyl acrylic esters with benzaldehyde and furfuraldehyde in the presence of DABCO, strongly depend upon the electronic and steric effects of the ester part. The “unreactivity” of acrylates increases with steric hindrance and with increasing chain length of the alcohol moiety in an acrylate [20,45]. The alcohol moiety in 4-acryloyloxy-2,2,6,6-tetramethylpiperidine-1-oxyl is undoubtly sterically hindered, so the expected reaction times will be long; however, this nitroxide has been chosen based on the availability of such nitroxyl esters. As an electrophilic partner in the MBH reaction, commonly used aldehydes were chosen, whose activity is broadly discussed in the literature. Aromatic aldehydes, especially those containing EWGs, are considered as reactive in the MBH reaction, in contrast to the aliphatic aldehydes (both *n*-butanal and pivalaldehyde), which are considered to be unreactive, although EWGs on the α-carbon atom (e.g., chloral) enhance their reactivity [20]. 2-Furaldehyde and nicotinic aldehyde were used because they were considered to be especially reactive in the MBH reaction [43].

## Results and Discussion

**Scheme 1:**
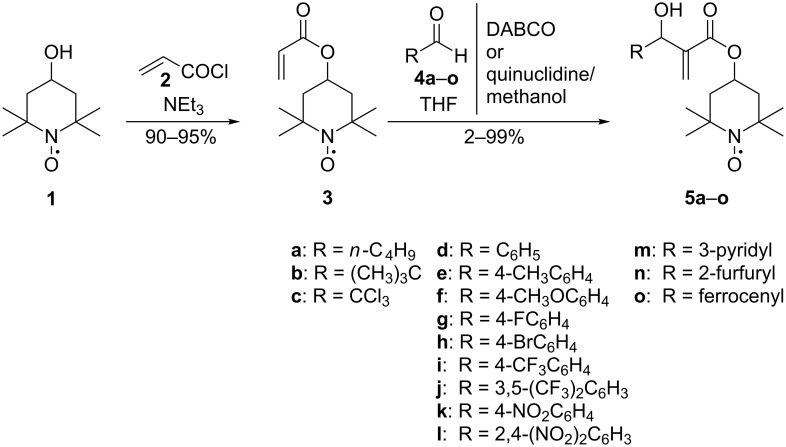
MBH adducts **5a**–**o** from 4-acryloyloxy-2,2,6,6-tetramethylpiperidine-1-oxyl.

4-Acryloyloxy-2,2,6,6-tetramethylpiperidine-1-oxyl (**3**) was obtained by esterification of acryloyl chloride (**2**) with 2,2,6,6-tetramethyl-4-piperidinol-1-oxyl (**1**) [46–47] in 90–95% yield. To synthesize MBH adducts (**5a**–**o**), **3** was reacted with aliphatic (**4a–c**), aryl (**4d–l**) and heterocyclic (**4m**,**n**) aldehydes, and (to obtain a compound bearing both nitroxyl and ferrocenyl moiety) ferrocenyl aldehyde (**4o**). Two catalytic systems were tested: DABCO, and quinuclidine with methanol as a cocatalyst. The latter system was chosen because it has been described as an excellent rate enhancer for the MBH reaction [9,11]. Methanol was chosen as an additive bearing a polar O–H bond, which activates both the aldehyde and “Michael” intermediate formed in the first step of the reaction, when an amine catalyst attacks an EWG activated olefin [11]. The reaction was carried out in THF as a solvent or the reagents were stirred neat. The synthesized MBH adducts (**5a**–**o**) are summarized in Scheme 1 and Table 1.

**Table 1:** Yields and melting points (mp) of MBH adducts **5a**–**o**.

**5**	R	DABCO *t* [h]	DABCO yield [%]	quinuclidine/methanol *t* [h]	quinuclidine/methanol yield [%]	mp [°C]

**a**	*n*-C_4_H_9_	333	36	96	81	83–85
**b**	(CH_3_)_3_C	330	—	480	33	red oil
**c**	CCl_3_	91	48	26	99	154–156
**d**	C_6_H_5_	115	78	48	92	116–118
**e**	4-CH_3_C_6_H_4_	672	—	336	42	130–133
**f**	4-CH_3_OC_6_H_4_	984	—	1112	27	121–124
**g**	4-FC_6_H_4_	1008	27	336	23	105–109
**h**	4-BrC_6_H_4_	672	24	168	56	127–130
**i**	4-CF_3_C_6_H_4_	768	76	72	77	132–135
**j**	3,5-(CF_3_)_2_C_6_H_3_	528	59	432	47	112–115
**k**	4-NO_2_C_6_H_4_	376	22	96	79	102–104
**l**	2,4-(NO_2_)_2_C_6_H_3_	90	69	30	56	50–57 (glass)
**m**	3-pyridyl	592	99	72	65	92–97
**n**	2-furyl	664	24	144	48	114–118
**o**	ferrocenyl	1128	—	1680	2	118–124

MBH adducts (**5a**–**o**) were obtained with very diverse results. Reaction times varied from one day to tens of days (**5f**,**g**,**o**). The reaction was significantly faster in the presence of quinuclidine/methanol system as a catalyst than in the presence of DABCO. The yields of the MBH adducts successfully obtained varied from negligible (**5o**, see below) to almost quantitative (**5c**,**m**). The use of 4-hydroxybenzaldehyde, 2-bromonicotinic aldehyde and 3-ferrocenylpropenal [48] did not result in any detectable MBH adducts in either catalytic system. The reaction with 2,4-dinitrobenzaldehyde (**4l**) provides some atypical intensely blue-colored side-products. The appropriate blue zones were isolated during column chromatography (Supporting Information File 1); however, attempts to obtain their physicochemical and spectral data were unsuccessful. The blue color of the side-products suggests that a nitroso group may be formed as a result of the reduction of the nitro group. Such transformations of 2,4-dinitrobenzaldehyde are well known. Under light, 2-nitroso-4-nitrobenzoic acid (characterized as its methyl ester) is formed [49]. In dilute aqueous sodium hydroxide solution, 4-nitroso-2-nitrophenol and formic acid are formed [50–52]. Ferrocenyl aldehyde (**4o**) as the starting compound [53] did not afford any product when DABCO was used as a catalyst. Use of the quinuclidine/methanol system and thorough searching of a potential product by TLC, resulted in the separation of the expected adduct **5o** in 2% yield. However, its identity was confirmed unambiguously by (HR-)MS (both EI and ESI) and IR spectroscopy. This poor result is probably caused by the cumulative steric hindrance of both ferrocenyl and nitroxyl moieties (the sensitivity of the MBH reaction to the steric hindrance of the alcohol moiety in an acrylate was commented on in the introduction [20,45]). Due to the radical nature of the adducts **5a**–**o**, their structures were confirmed by (HR-)MS (both EI and ESI), and IR spectroscopy (see Supporting Information File 1 for EIMS and IR spectra). Most of the EIMS spectra show the *m*/*z* 124 peak as the base one (except **5k**,**m**,**o**), and the abundant intensity of *m*/*z* 109 peak (except **5c**,**f**,**l**,**o**). The both fragments: *m*/*z* 124 and 109 are originated from the nitroxyl moiety of the investigated compounds **5**. HRMS–EI for *m*/*z* 124: calcd for C_9_H_16_: 124.12520; found: 124.12515; for *m*/*z* 109: calcd for C_8_H_13_: 109.10173; found: 109.10079. However, direct characterization of the adducts **5a**–**o** by NMR is impossible, one of the adducts (**5d**, R = C_6_H_5_) was subjected to the exemplary experiment developed for nitroxides by Keana and coworkers in 1975 [54]. **5d** was reduced in situ with phenylhydrazine directly in an NMR tube to a nonradical, corresponding *N*-hydroxylamine. The ^1^H NMR and ^13^C NMR spectra were recorded, but only their aliphatic part was found to be valuable due to the signals belonging to phenylhydrazine itself. The recorded spectral data are presented in the experimental part and the Supporting Information File 1 (together with spectra of phenylhydrazine itself, as a background) as well. The observed singlet in ^1^H NMR at δ 5.54, and symmetric narrow multiplets at 5.76–5.90 and 6.28–6.45 assigned to the C_6_H_5_–C**H**(OH)–C(=C**H_2_**)- fragment are well consistent with the spectra of the typical series of the MBH adducts of common aldehydes and methyl acrylate, presented in [32]. Synthesized compounds **5a**–**o** showed a weak antifungal activity. No insecticidal, acaricidal and herbicidal activity were shown.

## Conclusion

In conclusion, it has been demonstrated that the use of 4-acryloyloxy-2,2,6,6-tetramethylpiperidine-1-oxyl (**3**) as a starting compound allows us to obtain new MBH adducts **5a**–**o** bearing a nitroxyl moiety. The use of quinuclidine with methanol as a catalyst instead of DABCO decreases the time of the reaction. No influence of the radical nature of 4-acryloyloxy-2,2,6,6-tetramethylpiperidine-1-oxyl (**3**) on the reaction course was observed.

## Experimental

**General:** 2,2,6,6-Tetramethyl-4-hydroxypiperidin-1-oxyl (**1**) was synthesized by the oxidation of 2,2,6,6-tetramethyl-4-piperidinol with 30% hydrogen peroxide (76.5% yield, mp 71–73 °C), according to [55–57]. Liquid aldehydes were purified by using vacuum distillation. THF was distilled over sodium under argon in the presence of benzophenone as an indicator. The experiments were performed in a 5 mL round-bottom flask, equipped with a magnetic stirrer. Most of the MBH adducts were obtained as red solids. TLC was carried out on silica gel Merck Alurolle 5562, Alufolien 5554. Column chromatography was performed by using Merck 1.09385.1000 or Zeochem 60 hyd 40–63 μm (0.040–0.063 mm, 230–400 mesh). TLC visualisation: UV 254 nm light and/or iodine vapours. EIMS data were recorded by using AMD 604 and Agilent Technologies 5975 B mass spectrometers. HRMS–EI data were recorded by using an AMD 604 mass spectrometer. ESIMS and HRMS–ESI (positive ions, CH_3_OH as solvent) were recorded by using a Micromass LCT apparatus. IR (ν, cm^−1^) data were recorded by using an FT/IR Jasco 420 spectrophotometer. ^1^H and ^13^C NMR data were collected by using a Varian UNITYplus 200 spectrophotometer.

**4-Acryloyloxy-2,2,6,6-tetramethylpiperidine-1-oxyl (3):** 2,2,6,6-Tetramethyl-4-piperidinol-1-oxyl (**1**, 0.344 g, 2 mmol) in benzene (≈5 mL) was placed in a round bottomed flask of 50 mL capacity equipped with a magnetic stirrer. A solution of triethylamine (1.54 g, 15.3 mmol, 2.1–2.2 mL) in benzene (6 mL) was added dropwise from a pipette. A solution of acryloyl chloride (**2**, 0.187 g, 2.08 mmol, 170 μL) in benzene (3.5 mL) was added dropwise from a syringe at room temperature. The progress of the reaction was monitored by TLC (benzene/ethyl acetate 9:1, benzene/methanol 9:1). The reaction mixture was stirred at room temperature for 20 h, then the second portion of **2** (0.094 g, 1.04 mmol, 85 μL) in benzene (1.7 mL) was added dropwise from a syringe. The reaction mixture was stirred at room temperature for 1 h. Depending on the results of the TLC control of the progress of the reaction the third portion of **2** may be added (≈50 μL). After the reaction had been terminated, the precipitate of triethylamine hydrochloride was filtered off and the filtrate was concentrated under reduced pressure. The red residue was subjected to column chromatography (hexane/ethyl acetate 9:1 as a mobile phase). The red eluent was collected to give red crystals of 4-acryloyloxy-2,2,6,6-tetramethylpiperidine-1-oxyl (**3**), yield: 0.41–0.43 g (90–95%); mp 102–104 °C (lit. [46]: 102.5–103 °C, [58]: 102 °C, [59]: 99 °C ); EIMS *m*/*z* (% relative intensity): M^+^ 226 (16), 194 (10), 154 (15), 141 (7), 140 (17), 139 (21), 124 (84), 109 (95), 98 (6), 95 (8), 82 (21), 81 (20), 69 (11), 68 (20), 67 (19), 55 (100), 41 (34); HRMS–EI (*m*/*z*): calcd for C_12_H_20_NO_3_, 226.14432; found, 226.14401; IR (KBr): 1720 (C=O), 1635 (C=C) cm^−1^.

**MBH adducts 5; general procedure without solvent with an excess of aldehyde (5a**,**b**,**d**,**n):** 4-Acryloyloxy-2,2,6,6-tetramethylpiperidine-1-oxyl (**3**, 0.4 mmol), and a catalyst (about 10–20 mol % DABCO or quinuclidine), methanol (only if quinuclidine is used as catalyst) (10 μL), and an excess of freshly distilled aldehyde (**4a**, **4b**, **4d**, **4n**, ≈1 mL) were stirred under argon at room temperature for the time mentioned in Table 1. The progress of the reaction was monitored by TLC (hexane/ethyl acetate 9:1, benzene/ethyl acetate 9:1, benzene/methanol 9:1). The MBH adduct **5** was isolated by direct column chromatography of the reaction mixture by using an appropriate mobile phase (hexane/ethyl acetate 9:1, benzene/ethyl acetate 95:5, benzene/methanol 95:5) to afford the desired adduct **5**.

**MBH adducts 5; general procedure in THF (5c, e–m, o):** An appropriate aldehyde, freshly distilled and added with a syringe if liquid (0.8 mmol (**4e**, **4f**, **4g**, **4h**, **4i**, **4j**, **4m**), 0.6 mmol (**4c**), or 0.4 mmol (**4k**, **4l**, **4o**)), 4-acryloyloxy-2,2,6,6-tetramethylpiperidine-1-oxyl (**3**, 0.4 mmol), a catalyst (about 10–20 mol % DABCO or quinuclidine), methanol (only if quinuclidine is used as catalyst, 10 μL), and anhydrous THF (1.0–1.5 mL), were stirred under argon at room temperature for the time mentioned in Table 1. The progress of the reaction was monitored by TLC (hexane/ethyl acetate 9:1, benzene/ethyl acetate 9:1, benzene/methanol 9:1). THF was evaporated under reduced pressure. The residue was subjected to column chromatography by using an appropriate mobile phase (hexane/ethyl acetate 9:1, benzene/ethyl acetate 95:5, benzene/methanol 95:5) to afford the desired adduct **5**.

**4-(2-((*n*-Butyl)hydroxymethyl)acryloyloxy)-2,2,6,6-tetramethylpiperidine-1-oxyl (5a):** Yield (DABCO): 46 mg (36%); Yield (quinuclidine): 101 mg (81%); orange solid; mp 83–85 °C; EIMS *m*/*z* (% relative intensity): M^+^ 312 (10), 255 (7), 240 (10), 155 (45), 154 (23), 141 (11), 140 (35), 139 (17), 124 (100), 109 (69), 100 (18), 98 (9), 95 (30), 85 (10), 83 (44), 82 (19), 81 (16), 74 (14), 69 (31), 67 (17), 56 (18), 55 (29), 41 (32); HRMS–EI (*m*/*z*): calcd for C_17_H_30_NO_4_: 312.21748; found: 312.21674; mass spectrum (ESI, *m*/*z*, %) 335 (100, [M + Na]^+^) 140 (46); HRMS–ESI (*m*/*z*): calcd for C_19_H_26_NO_4_Na, 335.2073; found, 335.2067; IR (KBr): 1701 (C=O), 1633 (C=C) cm^−1^.

**4-(2-((*tert*-Butyl)hydroxymethyl)acryloyloxy)-2,2,6,6-tetramethylpiperidine-1-oxyl (5b):** Yield (quinuclidine): 41 mg (33%); red oil; EIMS *m*/*z* (% relative intensity): M^+^ 312 (11), 241 (6), 172 (7), 156 (44), 155 (64), 140 (73), 124 (100), 109 (65), 100 (49), 98 (17), 95 (22), 85 (13), 83 (22), 82 (27), 81 (24), 74 (55), 69 (46), 67 (23), 58 (17), 57 (98), 56 (38), 55 (42), 41 (65); HRMS–ESI (*m*/*z*): calcd for C_17_H_30_NO_4_, 312.21748; found, 312.21818; ESIMS *m*/*z* (% relative intensity): [M + Na]^+^ 335 (100); HRMS–ESI (*m*/*z*): [M + Na]^+^ calcd for C_17_H_30_NO_4_Na, 335.2073; found, 335.2042; IR (KBr): 1706 (C=O) 1627 (C=C) cm^−1^.

**4-(2-((Trichloromethyl)hydroxymethyl)acryloyloxy)-2,2,6,6-tetramethylpiperidine-1-oxyl (5c):** Yield (DABCO): 72 mg (48%); yield (quinuclidine): 148 mg (99%); yellow powder; mp 154–156 °C; EIMS *m*/*z* (% relative intensity): 374 (5), M^+^ 372 (5), 240 (9), 203 (4), 201 (5), 167 (9), 165 (15), 155 (24), 154 (20), 140 (17), 139 (19), 124 (100), 110 (6), 100 (10), 98 (6), 95 (5), 85 (11), 83 (7), 82 (17), 81 (14), 69 (22), 68 (11), 67 (13), 56 (14), 55 (20); HRMS–EI (*m*/*z*): calcd for C_14_H_21_NO_4_Cl_3_, 372.05362; found, 372.05425; ESIMS *m*/*z* (% relative intensity): 397 (86), [M + Na]^+^ 395 (100); HRMS–ESI (*m*/*z*): [M + Na]^+^ calcd for C_14_H_21_Cl_3_NO_4_Na, 395.0434; found, 395.0429; IR (KBr): 1710 (C=O), 1633 (C=C) cm^−1^.

**4-(2-((Phenyl)hydroxymethyl)acryloyloxy)-2,2,6,6-tetramethylpiperidine-1-oxyl (5d):** Yield (DABCO): 104 mg (78%); yield (quinuclidine): 122 mg (92%); orange crystals; mp 116–118 °C; EIMS *m*/*z* (% relative intensity): M^+^ 332 (9), 302 (3), 284 (2), 154 (16), 140 (11), 133 (8), 124 (100), 117 (28), 116 (14), 115 (27), 109 (82), 79 (21); HRMS–EI calcd for C_19_H_26_NO_4_, 332.1862; found, 332.1856; ESIMS *m*/*z* (% relative intensity): [M + Na]^+^ 355 (100); HRMS–ESI (*m*/*z*): [M + Na]^+^ calcd for C_19_H_26_NO_4_Na, 355.1760; found, 355.1742; IR (KBr): 1707 (C=O), 1628 (C=C) cm^−1^; ^1^H NMR (200 MHz, CDCl_3_, after reduction with phenylhydrazine in situ, in an NMR tube, in CDCl_3_, aliphatic part of the spectrum, in fact the spectrum of the corresponding hydroxylamine [54]) δ 1.16, 1.18, 1.19, 1.21 (4s, 12H, 4CH_3_), 1.40–1.64 (m, 2H, CH_2_), 1.75–1.95 (m, 2H, CH_2_), 4.96–5.18 (m, 1H, C**H**–OC(=O)), 5.54 (s, 1H, C**H**–OH), 5.76–5.90 (m, 1H, =C**H**H), 6.28–6.45 (m, 1H, =CH**H**) ppm; ^13^C NMR (50 MHz, CDCl_3_, after reduction with phenylhydrazine in situ, in an NMR tube, in CDCl_3_, aliphatic part of the spectrum, in fact the spectrum of the corresponding hydroxylamine [54]) δ 20.65 (2CH_3_), 32.00 (2CH_3_), 43.75 (CH_2_, confirmed by DEPT 135, piperidine ring), 43.81 (CH_2_, confirmed by DEPT 135, piperidine ring), 43.97 (CH_2_, confirmed by DEPT 135, piperidine ring), 59.48 (2C, absent in DEPT 135, piperidine ring), 67.68 (**C**HOC=O), 73.32 (CHOH), 126.12 (C=**C**H_2_, confirmed by DEPT 135), 126.84 (2CH_ar_), 128.03 (CH_ar_), 128.64 (2CH_ar_), 141.63 (**C_ar_**–CHOH, absent in DEPT 135), 142.50 (**C**=CH_2_, absent in DEPT 135), 165.98 (C=O, absent in DEPT 135) ppm.

**4-(2-((4-Methylphenyl)hydroxymethyl)acryloyloxy)-2,2,6,6-tetramethylpiperidine-1-oxyl (5e):** Yield (quinuclidine): 58 mg (42%); orange solid; mp 130–133 °C; EIMS *m*/*z* (% relative intensity): M^+^ 346 (22), 191 (13), 175 (15), 173 (12), 156 (11), 155 (10), 154 (22), 147 (9), 140 (18), 139 (16), 131 (25), 130 (9), 129 (15), 124 (100), 109 (67), 93 (11), 91 (19), 82 (13), 81 (14), 77 (9), 74 (8), 69 (13), 68 (7), 69 (13), 56 (8), 55 (13), 41 (14); HRMS–EI (*m*/*z*): calcd for C_20_H_28_NO_4_, 346.20183; found, 346.20093; ESIMS *m*/*z* (% relative intensity): [2M + Na] 715 (3), [M + Na]^+^ 369 (100); HRMS–ESI (*m*/*z*): [M + Na]^+^ calcd for C_20_H_28_NO_4_Na, 369.1905; found, 369.1916; IR (KBr): 1709 (C=O), 1630 (C=C) cm^−1^.

**4-(2-((4-Methoxyphenyl)hydroxymethyl)acryloyloxy)-2,2,6,6-tetramethylpiperidine-1-oxyl (5f):** Yield (quinuclidine): 39 mg (27%); orange solid; mp 121–124 °C; EIMS *m*/*z* (% relative intensity): M^+^ 362 (23), 208 (13), 207 (17), 191 (23), 190 (14), 189 (19), 173 (12), 163 (9), 162 (8), 154 (42), 147 (10), 146 (14), 145 (20), 140 (31), 139 (19), 137 (23), 135 (36), 124 (100), 110 (7), 69 (23), 57 (6), 56 (11), 41 (21); HRMS–EI (*m*/*z*): calcd for C_20_H_28_NO_5_, 362.19675; found, 362.19501; EIMS *m*/*z* (% relative intensity): [M + Na]^+^ 385 (100), 119 (15); HRMS–ESI (*m*/*z*): [M + Na]^+^ calcd for C_20_H_28_NO_5_Na, 385.1865; found, 385.1841; IR (KBr): 1707 (C=O), 1611 (C=C) cm^−1^.

**4-(2-((4-Fluorophenyl)hydroxymethyl)acryloyloxy)-2,2,6,6-tetramethylpiperidine-1-oxyl (5g):** Yield (DABCO): 38 mg (27%); Yield (quinuclidine): 33 mg (23%); yellow powder; mp 105–109 °C; EIMS *m*/*z* (% relative intensity): M^+^ 350 (13), 195 (5), 180 (5), 179 (7), 177 (4), 154 (33), 140 (22), 139 (16), 135 (25), 134 (12), 133 (20), 124 (100), 109 (83), 97 (15), 83 (6), 82 (16), 69 (25), 67 (13), 56 (11), 55 (18), 41 (22); HRMS–EI (*m*/*z*): calcd for C_19_H_25_NO_4_F, 350.17676; found, 350.17570; ESIMS *m*/*z* (% relative intensity): [M + Na]^+^ 373 (20), 288 (100); HRMS–ESI (*m*/*z*): [M + Na]^+^ calcd for C_19_H_25_FNO_4_Na, 373.1665; found: 373.1652; IR (KBr): 1712 (C=O), 1631 (C=C) cm^−1^.

**4-(2-((4-Bromophenyl)hydroxymethyl)acryloyloxy)-2,2,6,6-tetramethylpiperidine-1-oxyl (5h):** Yield (DABCO): 39 mg (24%); yield (quinuclidine): 92 mg (56%); orange powder; mp 127–130 °C; EIMS *m*/*z* (% relative intensity): 412 (12), M^+^ 410 (11), 255 (5), 239 (7), 185 (6), 160 (23), 154 (43), 140 (15), 139 (17), 124 (100), 116 (21), 109 (66), 69 (18), 55 (14), 41 (16); HRMS–EI (*m*/*z*): calcd for C_19_H_25_NO_4_Br, 410.09669; found, 410.09745; ESIMS *m*/*z* (% relative intensity): 435 (80), [M + Na]^+^ 433 (80), 414 (95), [M + 2H]^+^ 412 (100); HRMS–ESI (*m*/*z*): [M + 2H]^+^ calcd for C_19_H_27_NO_4_Br, 412.1123, found, 412.1109; HRMS–ESI (*m*/*z*): [M + Na]^+^ calcd for C_19_H_25_NO_4_BrNa, 433.0865; found, 433.0868; IR (KBr): 1708 (C=O), 1630 (C=C) cm^−1^.

**4-(2-((4-Trifluoromethylphenyl)hydroxymethyl)acryloyloxy)-2,2,6,6-tetramethylpiperidine-1-oxyl (5i):** Yield (DABCO): 122 mg (76%); yield (quinuclidine): 123 mg (77%); orange-yellow powder; mp 132–135 °C; EIMS *m*/*z* (% relative intensity): M^+^ 400 (13), 229 (6), 201 (10), 185 (15), 184 (8), 183 (12), 154 (39), 141 (7), 139 (16), 127 (16), 125 (15), 124 (100), 109 (82), 85 (10), 83 (6), 82 (14), 81 (16), 74 (9), 69 (22), 68 (10), 67 (12), 57 (7), 56 (11), 55 (17), 41 (22); HRMS–EI (*m*/*z*): calcd for C_20_H_25_NO_4_F_3_, 400.17357; found, 400.17225; ESIMS *m*/*z* (% relative intensity): [M + Na]^+^ 423 (100); HRMS–ESI (*m*/*z*): [M + Na]^+^ calcd for C_20_H_25_F_3_NO_4_Na, 423.1633; found, 423.1629; IR (KBr): 1712 (C=O), 1630 (C=C) cm^−1^.

**4-(2-((3,5-Bis(trifluoromethyl)phenyl)hydroxymethyl)acryloyloxy)-2,2,6,6-tetramethylpiperidine-1-oxyl (5j):** Yield (DABCO): 111 mg (59%); yield (quinuclidine): 88 mg (47%); light-orange crystals; mp 112–115 °C; EIMS *m*/*z* (% relative intensity): M^+^ 468 (16), 454 (4), 434 (10), 253 (10), 252 (5), 251 (4), 249 (6), 243 (6), 241 (9), 233 (10), 229 (8), 213 (7), 195 (13), 154 (36), 140 (22), 139 (17), 124 (100), 109 (74), 85 (12), 82 (14), 81 (14), 69 (15), 68 (9), 67 (11), 57 (6), 56 (10), 55 (14), 41 (16); HRMS–EI (*m*/*z*): calcd for C_21_H_24_NO_4_F_6_, 468.16095; found, 468.16152; ESIMS *m*/*z* (% relative intensity): [M + Na]^+^ 491 (100); HRMS–ESI (*m*/*z*): [M + Na]^+^ calcd for C_21_H_24_F_6_NO_4_Na, 491.1507; found: 491.1459; IR (KBr): 1714 (C=O), 1638 (C=C) cm^−1^.

**4-(2-((4-Nitrophenyl)hydroxymethyl)acryloyloxy)-2,2,6,6-tetramethylpiperidine-1-oxyl** (**5k):** Yield (DABCO): 33 mg (22%); yield (quinuclidine): 120 mg (79%); yellow powder; mp 102–104 °C; EIMS *m*/*z* (% relative intensity): M^+^ 377 (15), 363 (12), 189 (8), 160 (25), 154 (51), 140 (67), 139 (19), 124 (76), 109 (100), 85 (13), 82 (22), 81 (21), 69 (29), 68 (13), 67 (16), 57 (9), 56 (16), 55 (23), 41 (28); HRMS–EI (*m*/*z*): calcd for C_19_H_25_N_2_O_6_, 377.17126; found, 377.17090; ESIMS *m*/*z* (% relative intensity): [M + Na]^+^ 400 (100); HRMS–ESI (*m*/*z*): [M + Na]^+^ calcd for C_19_H_25_N_2_O_6_Na, 400.1610; found, 400.1603; IR (KBr): 1713 (C=O), 1636 (C=C), 1521 (NO_2_), 1351 (NO_2_) cm^−1^.

**4-(2-((2,4-Dinitrophenyl)hydroxymethyl)acryloyloxy)-2,2,6,6-tetramethylpiperidine-1-oxyl (5l):** Yield (DABCO): 117 mg (69%); yield (quinuclidine): 95 mg (56%); red glass; mp 50–67 °C; EIMS *m*/*z* (% relative intensity): M^+^ 422 (8), 408 (5), 179 (8), 154 (34), 140 (34), 139 (15), 124 (100), 85 (10), 82 (15), 81 (15), 69 (21), 68 (9), 67 (13), 57 (10), 56 (13), 55 (21), 41 (22); HRMS–EI (*m*/*z*): calcd for C_19_H_24_N_3_O_8,_ 422.15634; found, 422.15561; ESIMS *m*/*z* (% relative intensity): [M + 2H]^+^ 424 (100); HRMS–ESI (*m*/*z*): [M + 2H]^+^ calcd for C_19_H_26_N_3_O_8_, 424.1720; found, 424.1729; IR (KBr): 1717 (C=O), 1628, (C=C), 1537 (NO_2_), 1347 (NO_2_) cm^−1^.

**4-(2-((3-Pyridyl)hydroxymethyl)acryloyloxy)-2,2,6,6-tetramethylpiperidine-1-oxyl (5m):** Yield (DABCO): 132 mg (99%); yield (quinuclidine): 87 mg (65%); orange-yellow powder; mp 92–97 °C; EIMS *m*/*z* (% relative intensity): 334 (12), M^+^ 333 (17), 303 (6), 301 (7), 247 (9), 180 (100), 163 (7), 162 (13), 154 (15), 144 (8), 140 (24), 135 (17), 124 (59), 118 (23), 117 (18), 109 (73), 82 (11), 81 (13), 80 (13), 79 (6), 78 (7), 69 (19), 68 (8), 67 (14), 56 (11), 55 (20), 53 (9), 41 (26); HRMS–EI (*m*/*z*): calcd for C_18_H_25_N_2_O_4_, 333.18143; found, 333.18050; ESIMS *m*/*z* (% relative intensity): [M + Na]^+^ 356 (100); HRMS–ESI (*m*/*z*): [M + Na]^+^ calcd for C_18_H_25_N_2_O_4_Na, 356.1712; found, 356.1708; IR (film) 1714 (C=O), 1632 (C=C) cm^−1^.

**4-(2-((2-Furyl)hydroxymethyl)acryloyloxy)-2,2,6,6-tetramethylpiperidine-1-oxyl (5n):** Yield (DABCO): 31 mg (24%); yield (quinuclidine): 62 mg (48%); dark orange-yellow powder; mp 114–118 °C; EIMS *m*/*z* (% relative intensity): M^+^ 322 (20), 305 (4), 154 (25), 151 (29), 140 (30), 124 (100), 109 (84), 97 (24), 83 (9), 82 (17), 81 (17), 69 (43), 67 (19), 56 (13), 55 (19), 41 (36); HRMS–EI (*m*/*z*): calcd for C_17_H_24_NO_5_, 322.16545; found, 322.16457; ESIMS *m*/*z* (% relative intensity): 346 (50), [M + Na]^+^ 345 (100); HRMS–ESI (*m*/*z*): [M + Na]^+^ calcd for C_17_H_24_NO_5_Na, 345.1552; found, 345.1554; IR (KBr): 1706 (C=O), 1633 (C=C) cm^−1^.

**4-(2-((Ferrocenyl)hydroxymethyl)acryloyloxy)-2,2,6,6-tetramethylpiperidine-1-oxyl (5o):** Yield (quinuclidine): 4 mg (2%); orange solid; mp 118–124 °C; EIMS *m*/*z* (% relative intensity): 441 (8), M^+^ 440 (16), 439 (1), 438 (2), 426 (6), 425 (14), 424 (2), 423 (7), 410 (2), 409 (4), 392 (3), 286 (64), 284 (9), 270 (14), 269 (6), 268 (7), 243 (8), 241 (5), 224 (25), 186 (7), 185 (9), 149 (18), 98 (35), 97 (22), 80 (78), 71 (34), 70 (15), 69 (31), 58 (39), 57 (64), 56 (18), 55 (52), 45 (13), 44 (67), 43 (36), 42 (22), 41 (48), 40 (100); HRMS–EI (*m*/*z*): calcd for C_23_H_30_NO_4_Fe; 440.15242; found, 440.15327; ESIMS *m*/*z* (% relative intensity): [M + Na]^+^ 463 (95), M^+^ 440 (100); HRMS–ESI (*m*/*z*): calcd for C_23_H_30_NO_4_Fe, 440.1524; found, 440.1495; IR (KBr): 1701 (C=O), 1633 (C=C) cm^−1^.

## Supporting Information

Supporting Information features EIMS and IR spectra of the synthesized compounds **5a–o**, ^1^H and ^13^C NMR of **5d** with phenylhydrazine, and the chromatographic separation of **5l**.

File 1 Detailed spectrographic data.
