# Supplementary material for: Reactions of nitroxides XIII: Synthesis of the Morita–Baylis–Hillman adducts bearing a nitroxyl moiety using 4-acryloyloxy-2,2,6,6-tetramethylpiperidine-1-oxyl as a starting compound, and DABCO and quinuclidine as catalysts
Source: Beilstein J Org Chem. 2012 Sep 12;8:1515–22. doi: 10.3762/bjoc.8.171 (PMC3458776; doi:10.3762/bjoc.8.171)

Supporting Information

for

Reactions of nitroxides XIII: Synthesis of the Morita–Baylis–Hillman adducts bearing a nitroxyl moiety using 4-acryloyloxy-2,2,6,6-tetramethylpiperidine-1-oxyl as a starting compound, and DABCO and quinuclidine as catalysts

Jerzy Zakrzewski

Address: Institute of Industrial Organic Chemistry, Annopol 6, 03-236 Warsaw, Poland

Email: Jerzy Zakrzewski - zakrzewski@ipo.waw.pl

Detailed spectrographic data

3, 4-Acryloyloxy-2,2,6,6-tetramethylpiperidine-1-oxyl, EIMS

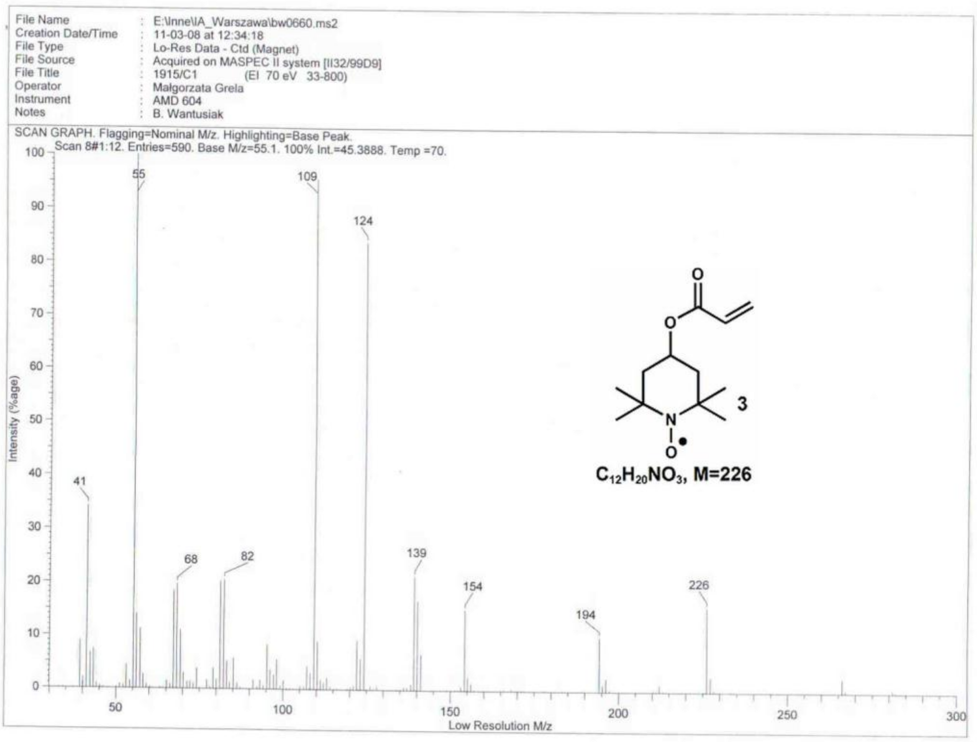

3, 4-Acryloyloxy-2,2,6,6-tetramethylpiperidine-1-oxyl, IR

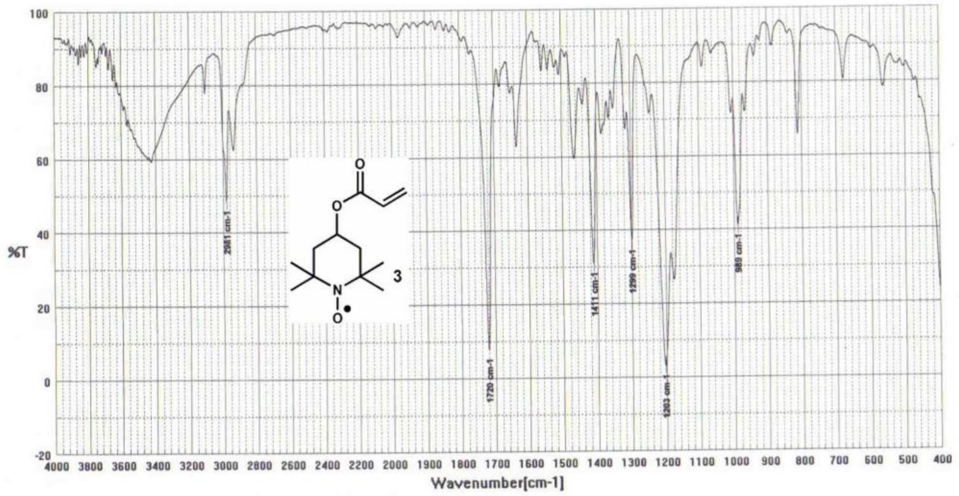

Sample Name	1891/C1 (0,6mg/270mg KBr)
Resolution	1 cm-1
Accumulation	30
Apodization	Cosine
Date/Time	109-06-15 12:42

5a, 4-(2-(*n*-Butylhydroxymethyl)acryloyloxy)-2,2,6,6-tetramethylpiperidine-1-oxyl, R=*n*-C_4H_9, EIMS

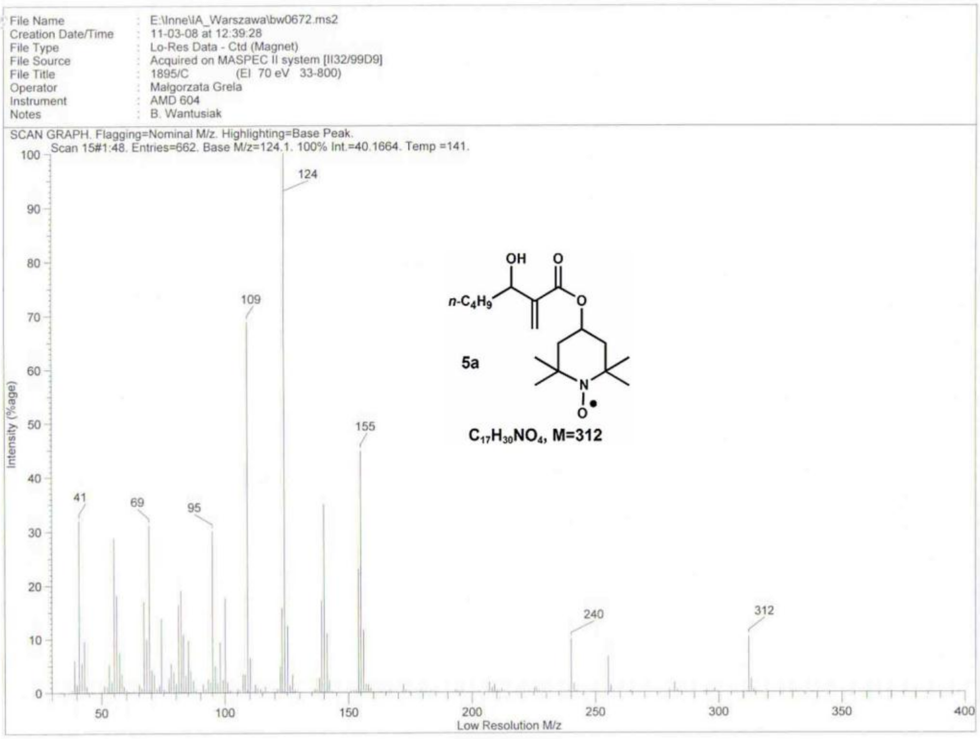

5a, 4-(2-(*n*-Butylhydroxymethyl)acryloyloxy)-2,2,6,6-tetramethylpiperidine-1-oxyl, R=*n*-C_4H_9, IR

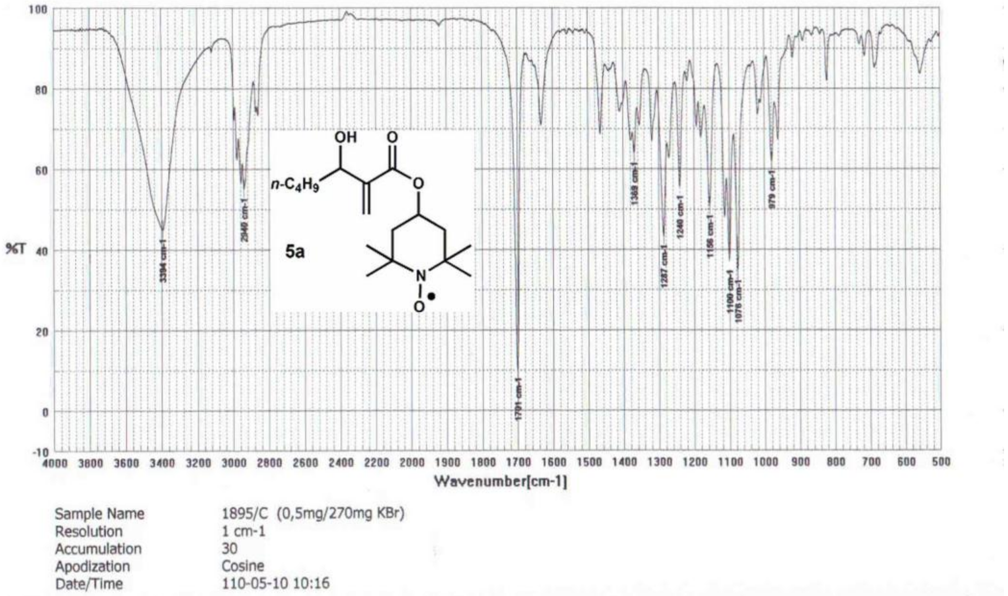

5b, 4-(2-(*t*-Butylhydroxymethyl)acryloyloxy)-2,2,6,6-tetramethylpiperidine-1-oxyl, R=*t*-C_4H_9, EIMS

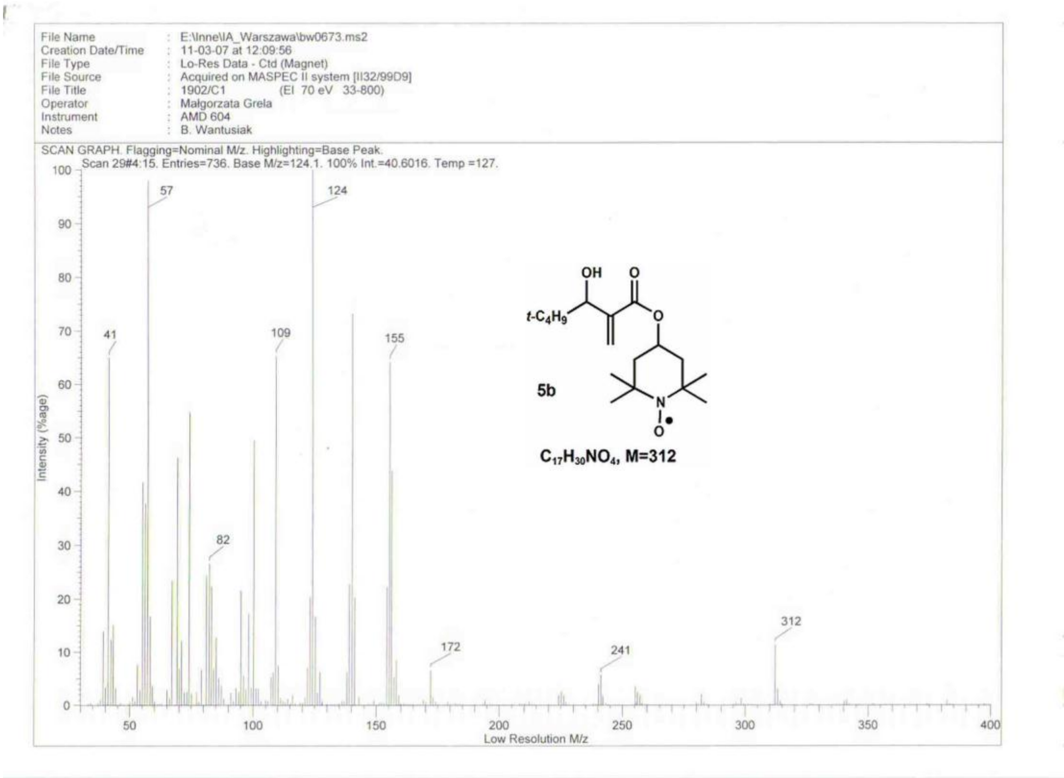

5b, 4-(2-(*t*-Butylhydroxymethyl)acryloyloxy)-2,2,6,6-tetramethylpiperidine-1-oxyl, R=*t*-C_4H_9, IR

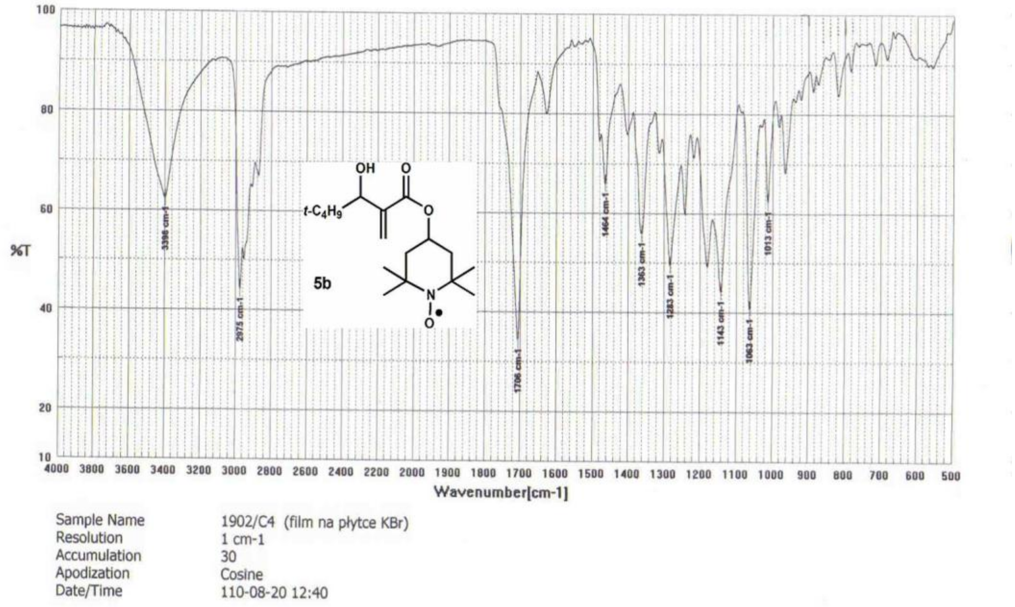

5c, 4-(2-(Trichloromethylhydroxymethyl)acryloyloxy)-2,2,6,6-tetramethylpiperidine-1-oxyl, R=CCl_3, EIMS

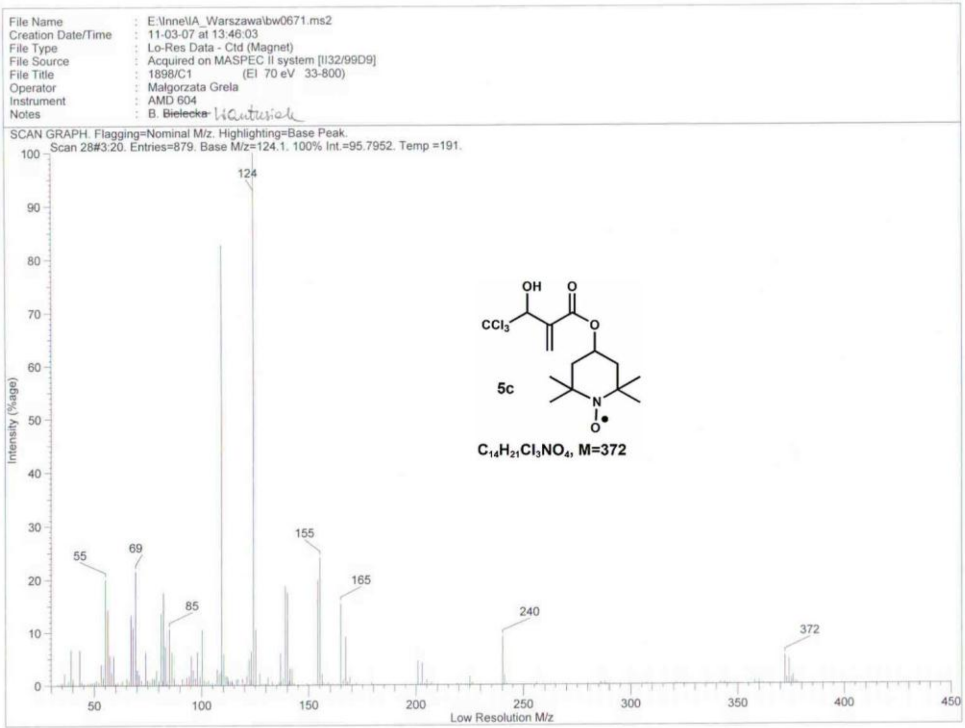

5c, 4-(2-(Trichloromethylhydroxymethyl)acryloyloxy)-2,2,6,6-tetramethylpiperidine-1-oxyl, R=CCl_3, IR

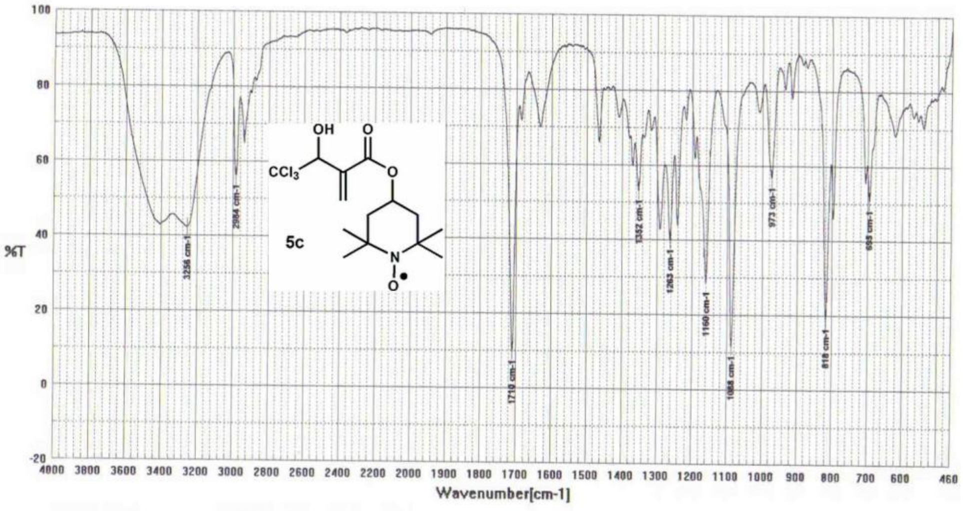

5d, 4-(2-(Phenylhydroxymethyl)acryloyloxy)-2,2,6,6-tetramethylpiperidine-1-oxyl, R=C_6H_5, EIMS

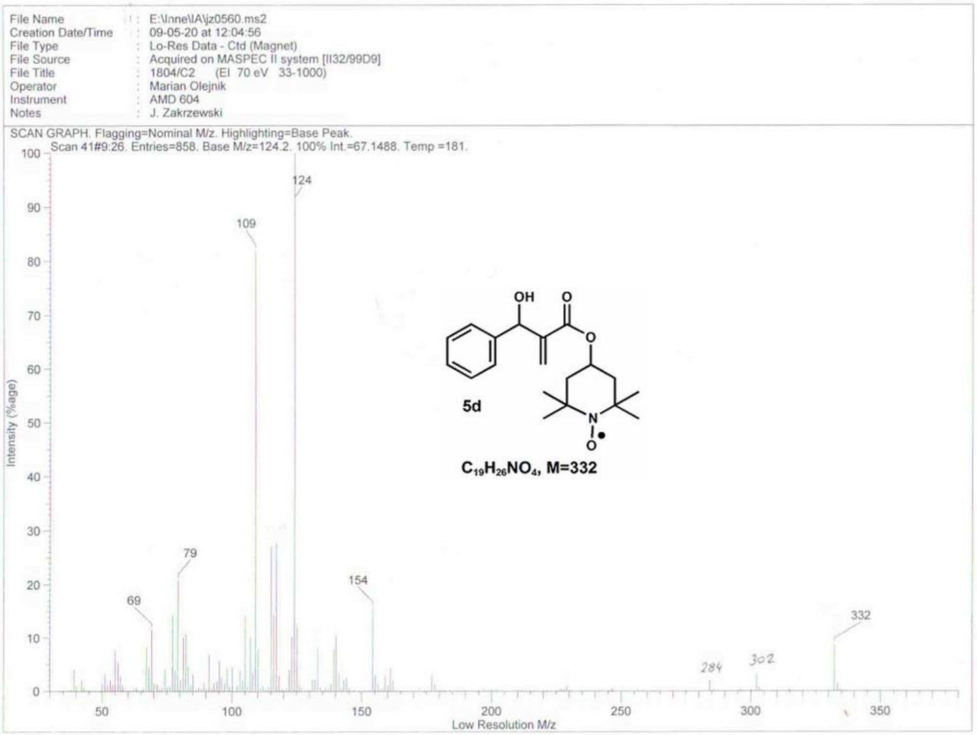

5d, 4-(2-(Phenylhydroxymethyl)acryloyloxy)-2,2,6,6-tetramethylpiperidine-1-oxyl, R=C_6H_5, IR

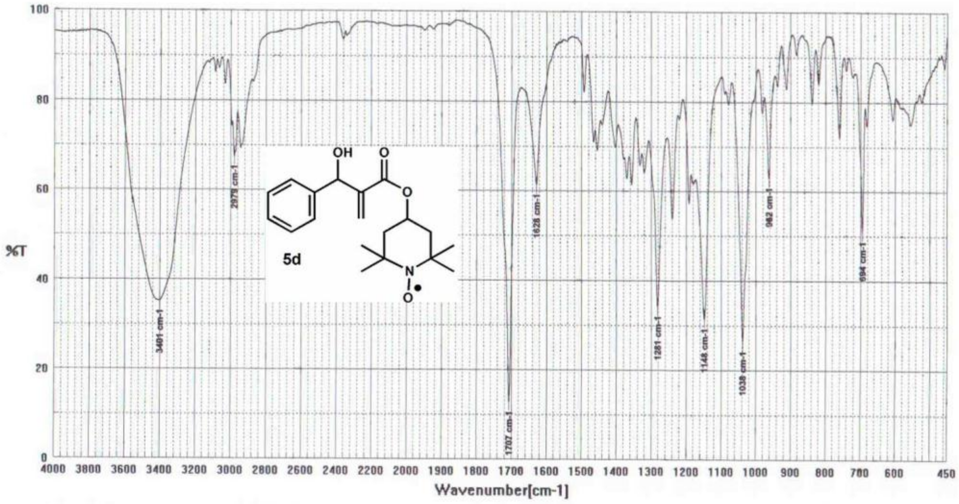

Sample Name	1804/C2 (0,5mg/270mg KBr)
Resolution	1 cm-1
Accumulation	30
Apodization	Cosine
Date/Time	109-02-17 11:31

5d, 4-(2-(Phenylhydroxymethyl)acryloyloxy)-2,2,6,6-tetramethylpiperidine-1-oxyl, R=C_6H_5;
^{1}H NMR: **5d** reduced *in situ* with $C_6H_5NHNH_2$ in an NMR tube

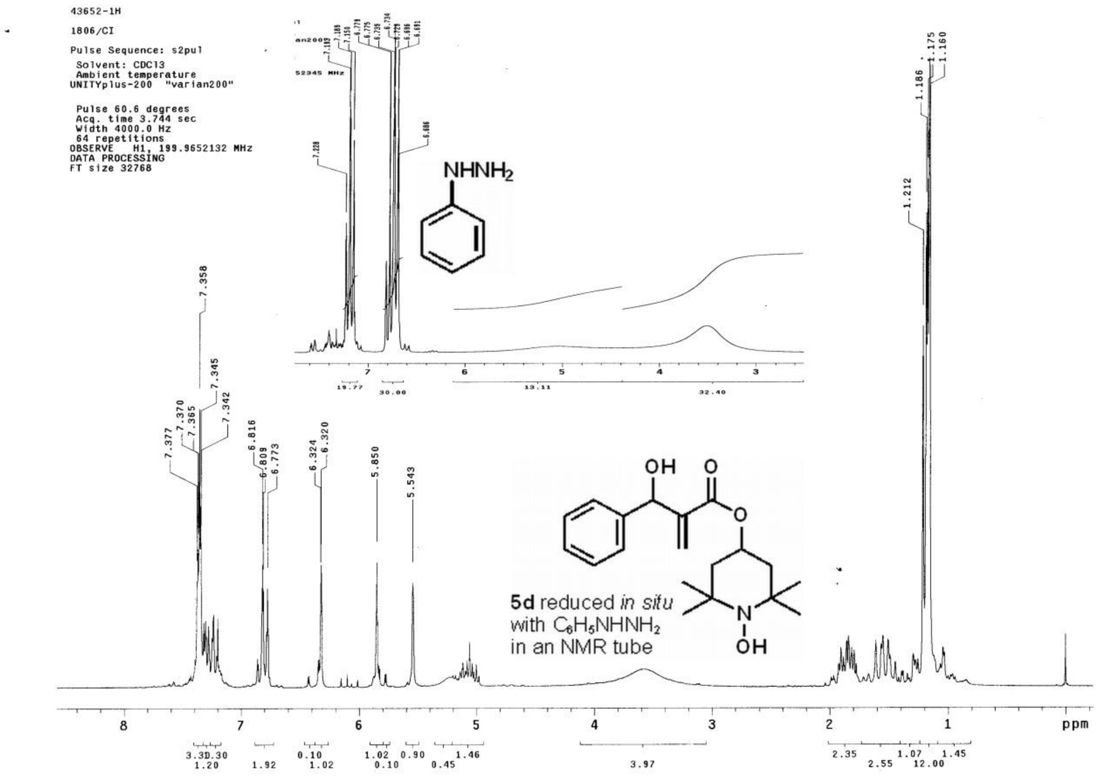

5d, 4-(2-(Phenylhydroxymethyl)acryloyloxy)-2,2,6,6-tetramethylpiperidine-1-oxyl, R=C_6H_5;
^{13}C NMR: **5d** reduced *in situ* with $C_6H_5NHNH_2$ in an NMR tube

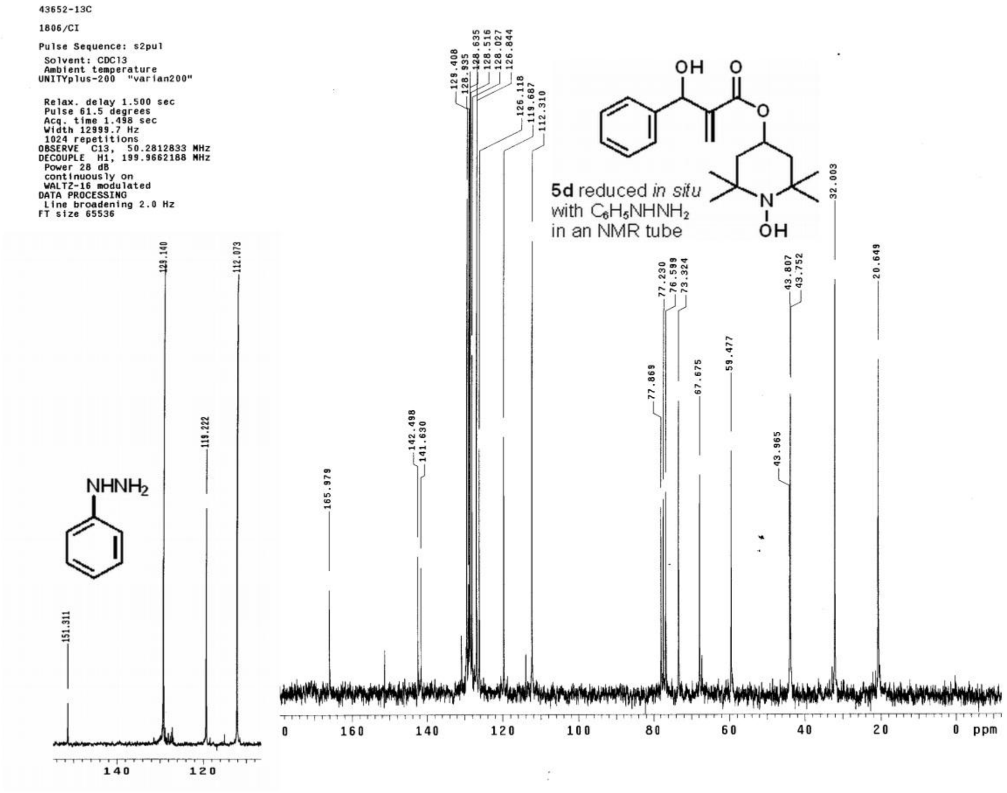

5d, 4-(2-(Phenylhydroxymethyl)acryloyloxy)-2,2,6,6-tetramethylpiperidine-1-oxyl, R=C_6H_5;
^{13}C NMR, DEPT 135°: **5d** reduced *in situ* with $C_6H_5NHNH_2$ in an NMR tube

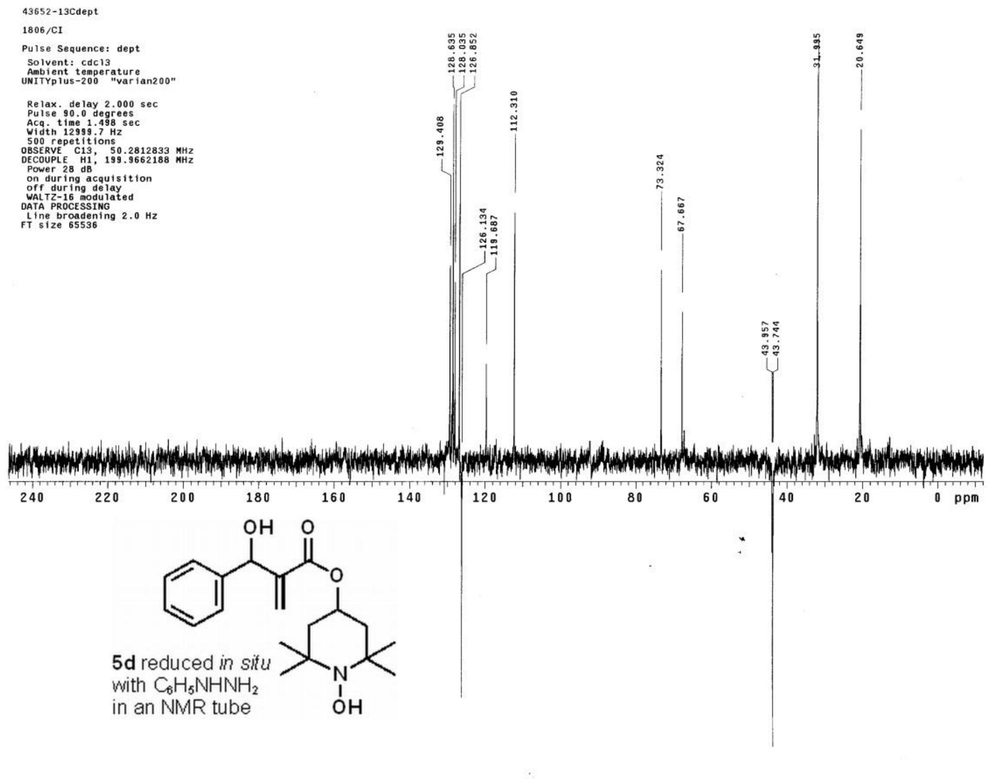

5e, 4-(2-((4-Methylphenyl)hydroxymethyl)acryloyloxy)-2,2,6,6-tetramethylpiperidine-1-oxyl, R=4-$CH_3C_6H_4$, EIMS

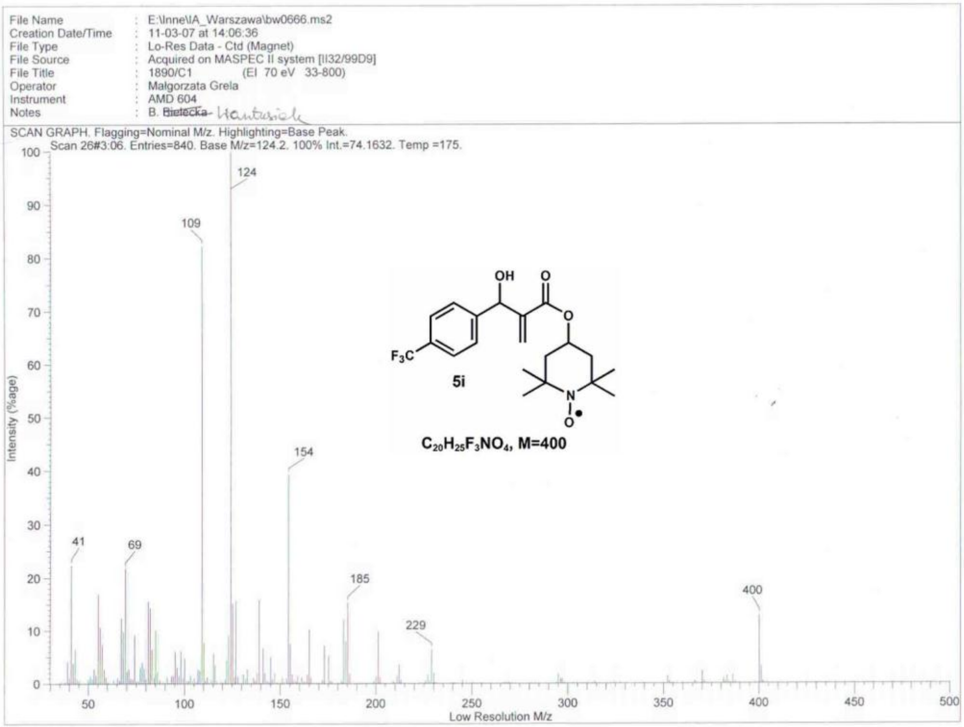

5e, 4-(2-((4-Methylphenyl)hydroxymethyl)acryloyloxy)-2,2,6,6-tetramethylpiperidine-1-oxyl, R=4-$CH_3C_6H_4$, IR

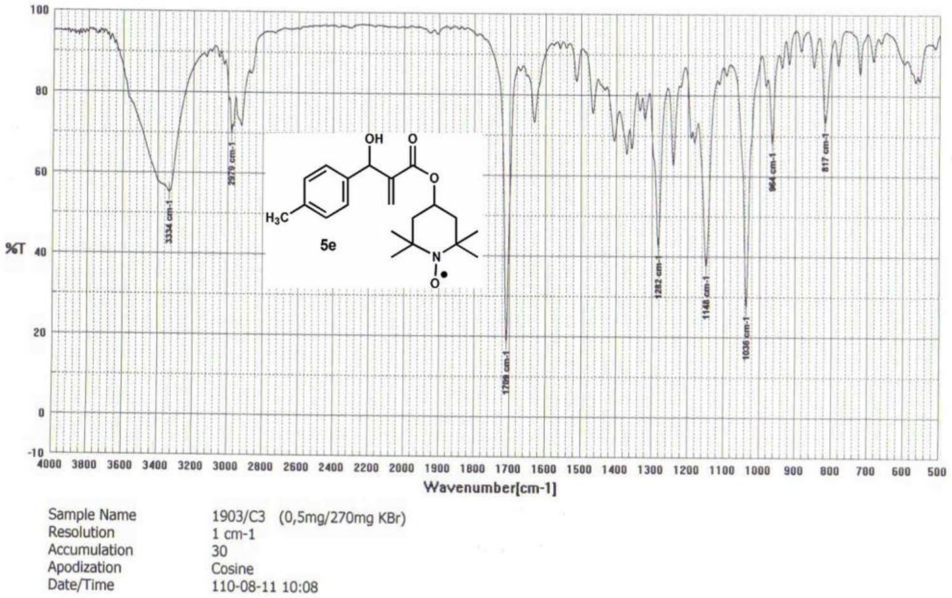

5f, 4-(2-((4-Methoxyphenyl)hydroxymethyl)acryloyloxy)-2,2,6,6-tetramethylpiperidine-1-oxyl, R=4-$CH_3OC_6H_4$, EIMS

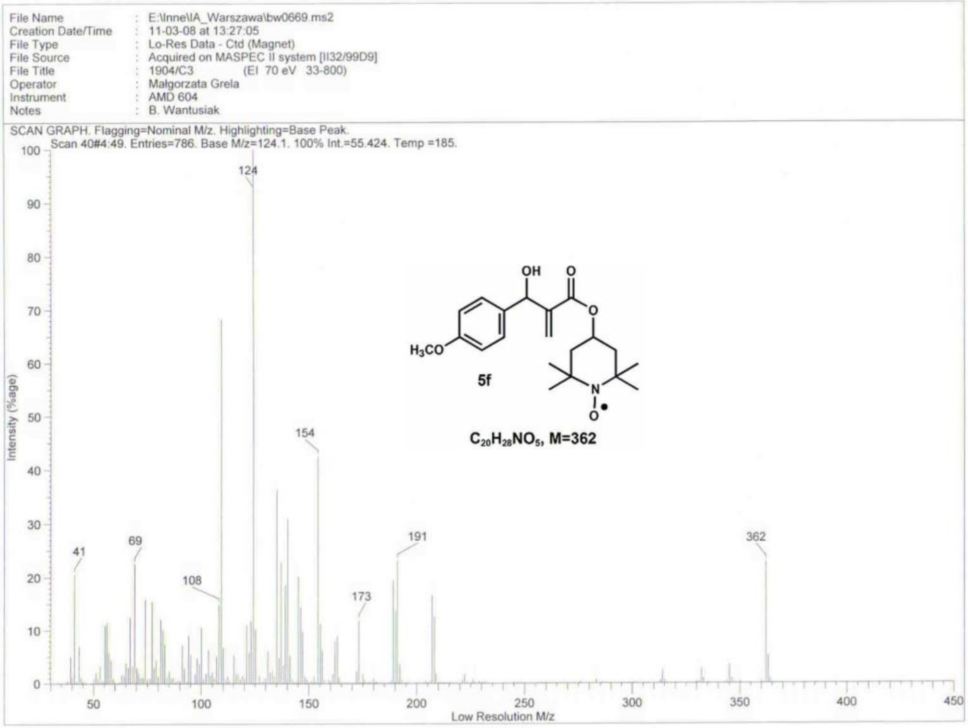

5f, 4-(2-((4-Methoxyphenyl)hydroxymethyl)acryloyloxy)-2,2,6,6-tetramethylpiperidine-1-oxyl, R=4-$CH_3OC_6H_4$, IR

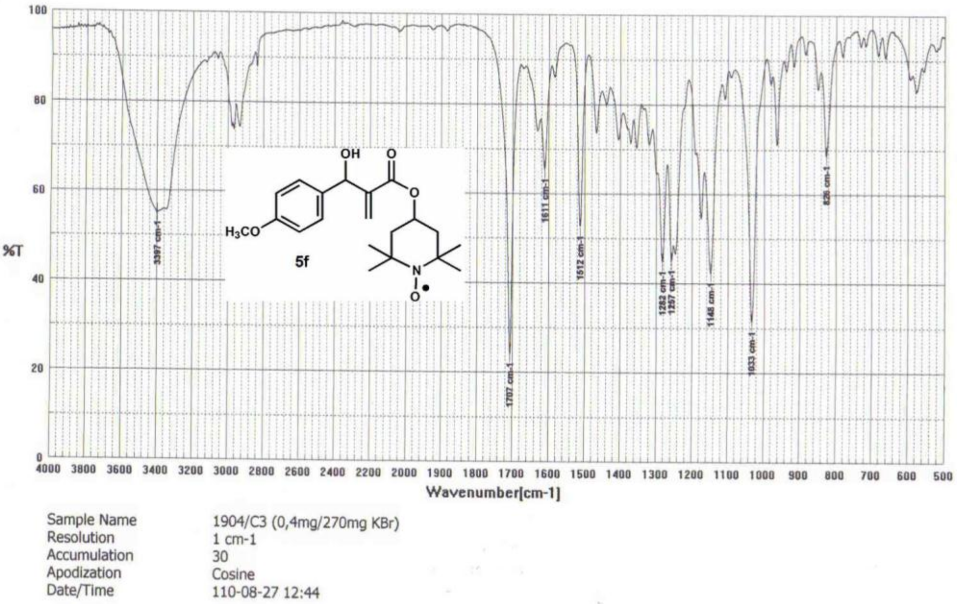

5g, 4-(2-((4-Fluorophenyl)hydroxymethyl)acryloyloxy)-2,2,6,6-tetramethylpiperidine-1-oxyl, R=4-FC_6H_4, EIMS

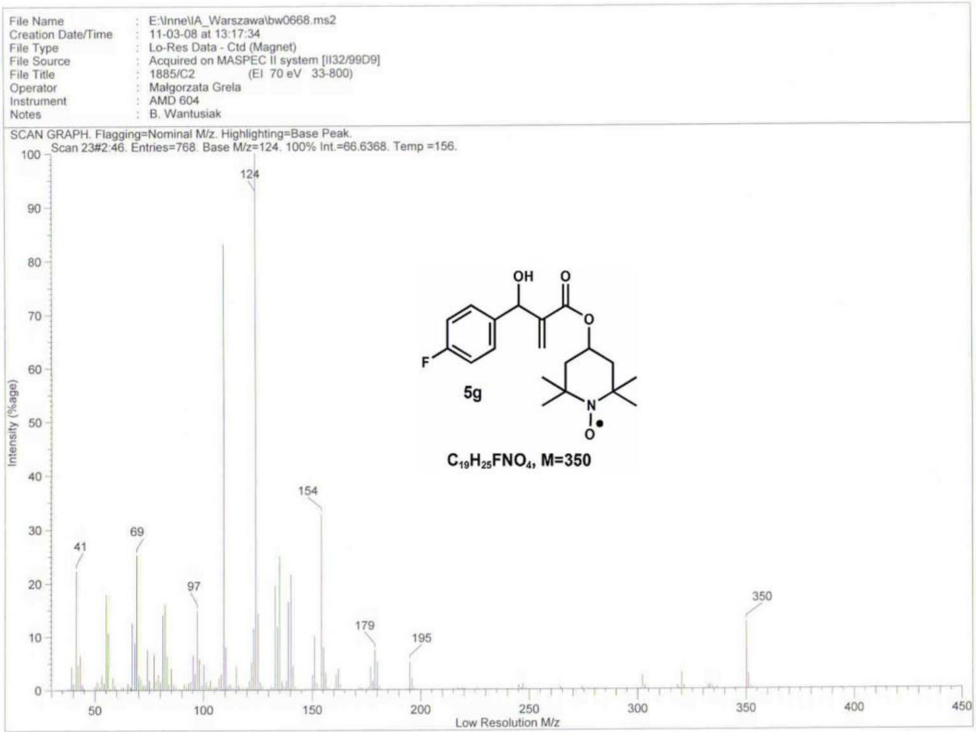

5g, 4-(2-((4-Fluorophenyl)hydroxymethyl)acryloyloxy)-2,2,6,6-tetramethylpiperidine-1-oxyl, R=4-FC_6H_4, IR

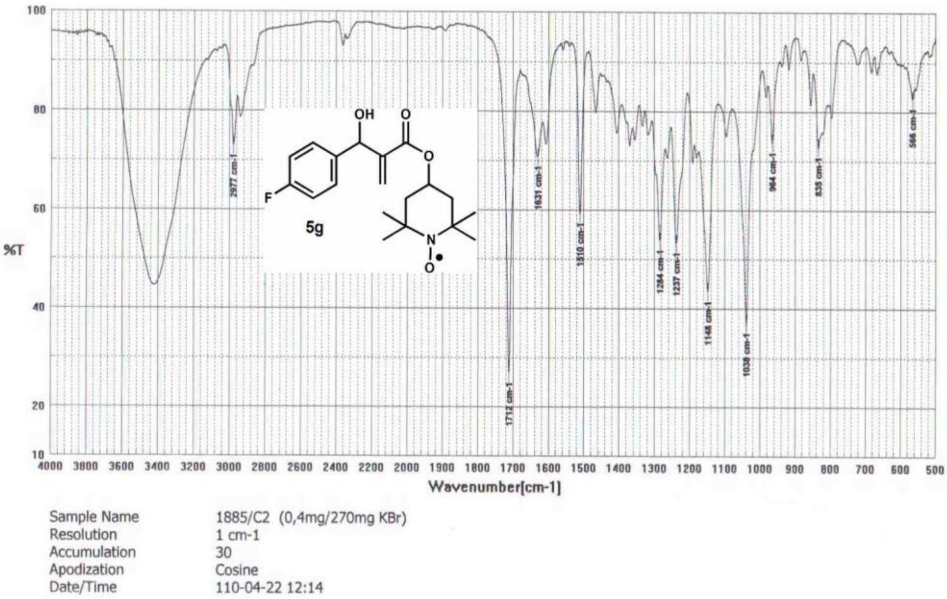

5h, 4-(2-((4-Bromophenyl)hydroxymethyl)acryloyloxy)-2,2,6,6-tetramethylpiperidine-1-oxyl, R=4-BrC_6H_4, EIMS

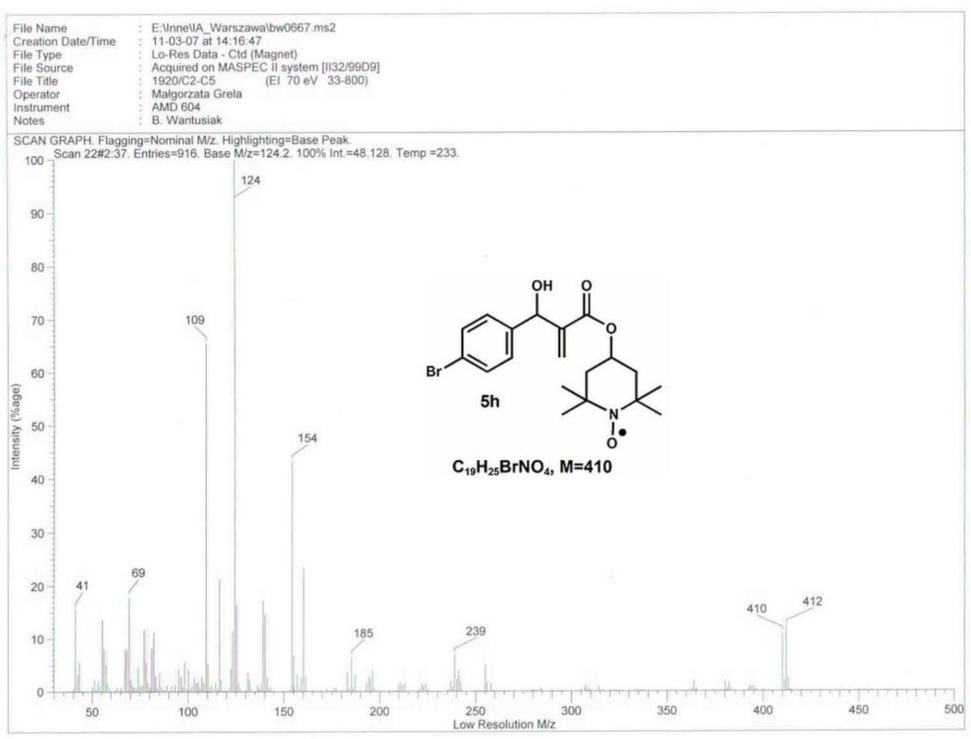

5h, 4-(2-((4-Bromophenyl)hydroxymethyl)acryloyloxy)-2,2,6,6-tetramethylpiperidine-1-oxyl, R=4-BrC_6H_4, IR

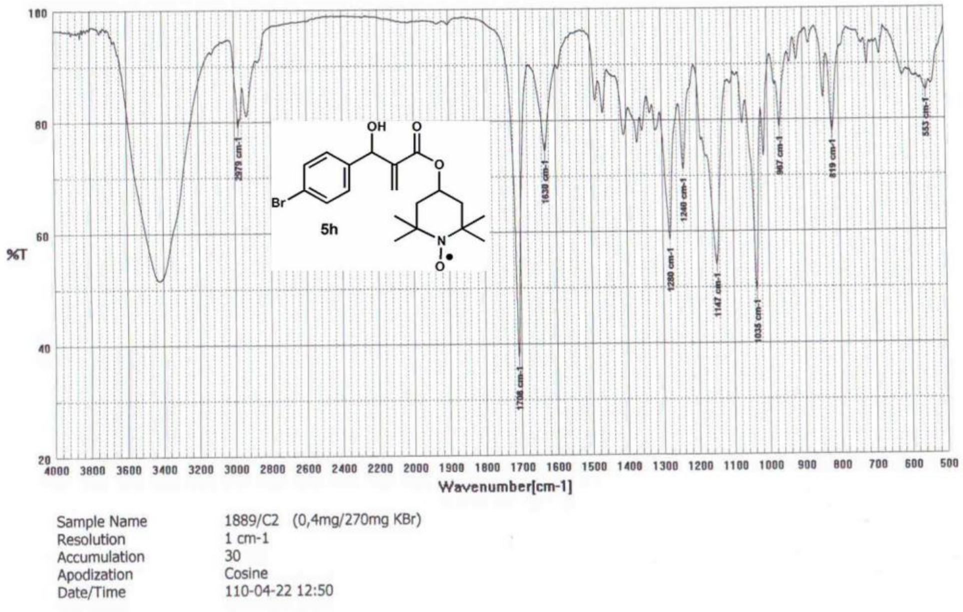

5i, 4-(2-((4-Trifluoromethylphenyl)hydroxymethyl)acryloyloxy)-2,2,6,6-tetramethylpiperidine-1-oxyl, R=4-$CF_3C_6H_4$, EIMS

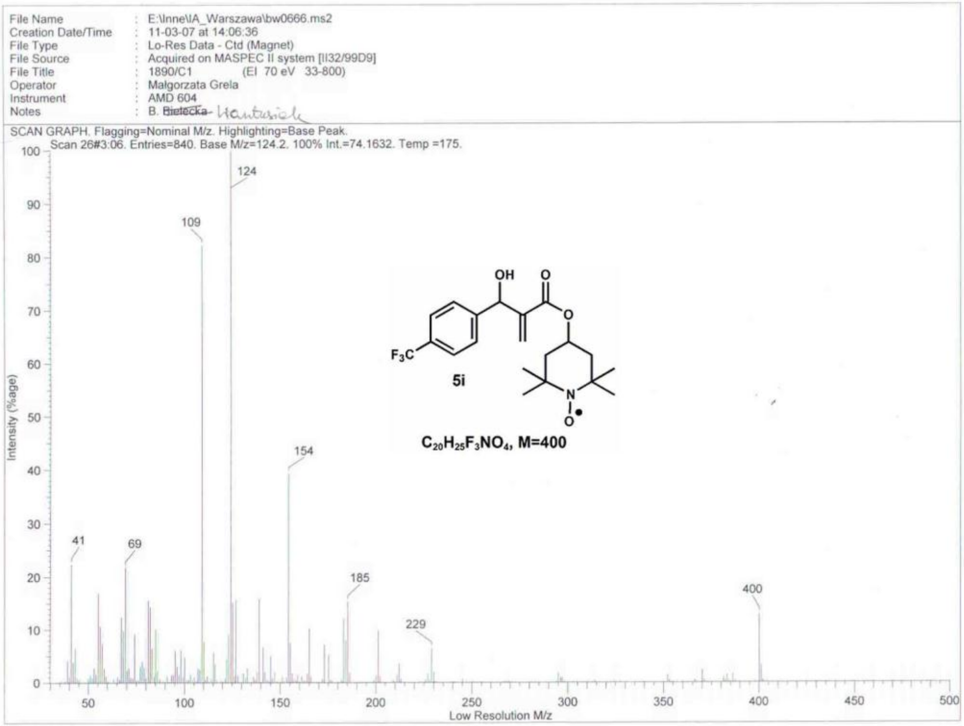

5i, 4-(2-((4-Trifluoromethylphenyl)hydroxymethyl)acryloyloxy)-2,2,6,6-tetramethylpiperidine-1-oxyl, R=4-$CF_3C_6H_4$, IR

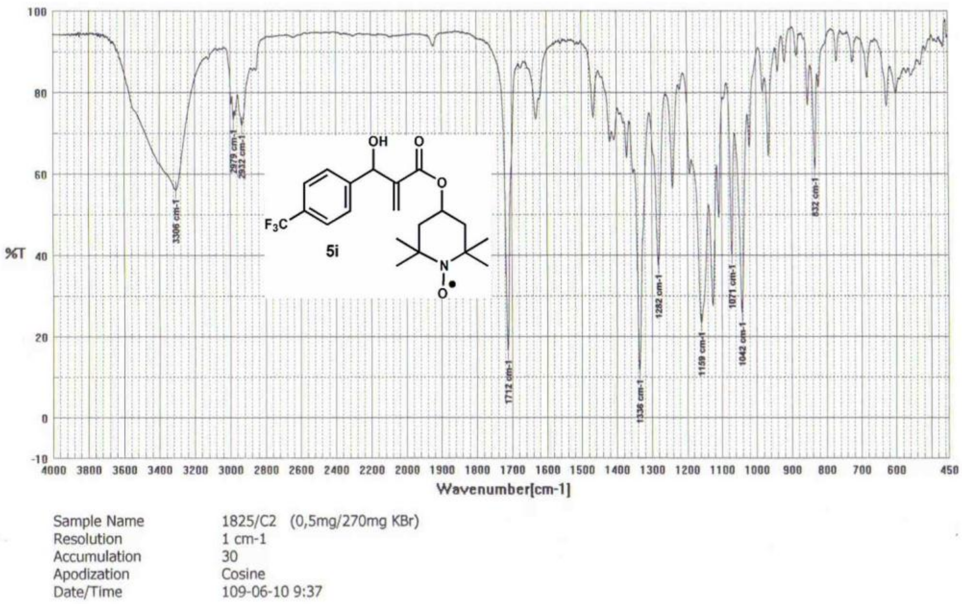

5j, 4-(2-((3,5-Bis(trifluoromethyl)phenyl)hydroxymethyl)acryloyloxy)-2,2,6,6-tetramethylpiperidine-1-oxyl, R=3,5-di-$CF_3C_6H_3$, EIMS

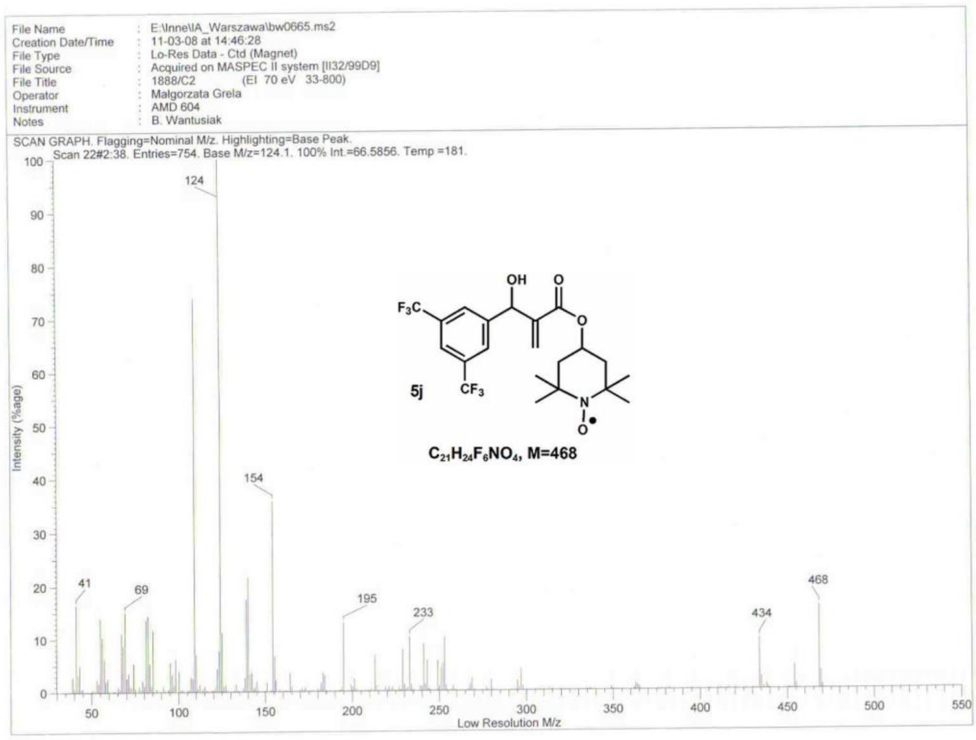

5j, 4-(2-((3,5-Bis(trifluoromethyl)phenyl)hydroxymethyl)acryloyloxy)-2,2,6,6-tetramethylpiperidine-1-oxyl, R=3,5-di-$CF_3C_6H_3$, IR

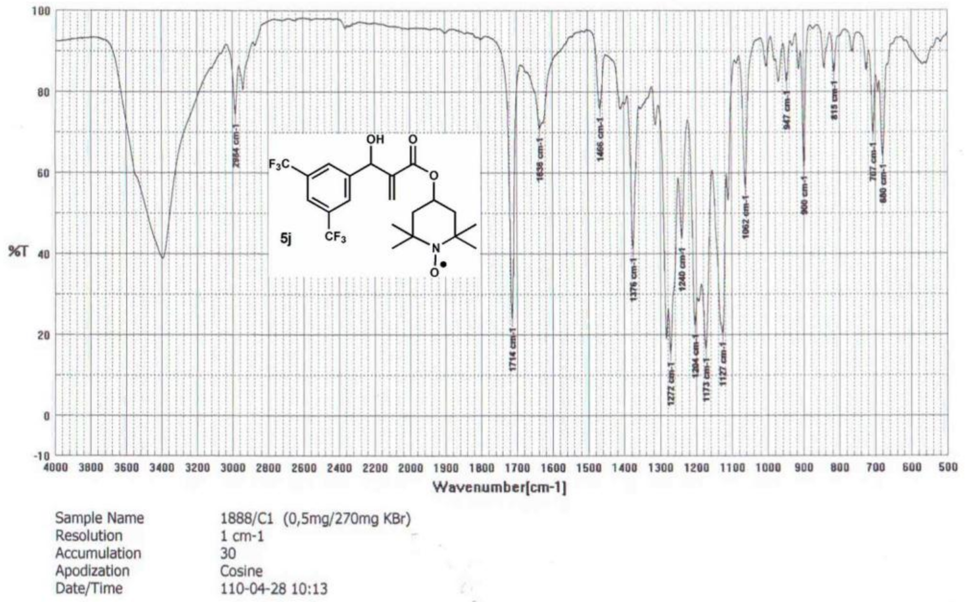

5k, 4-(2-((4-Nitrophenyl)hydroxymethyl)acryloyloxy)-2,2,6,6-tetramethylpiperidine-1-oxyl, R=4-$NO_2C_6H_4$, EIMS

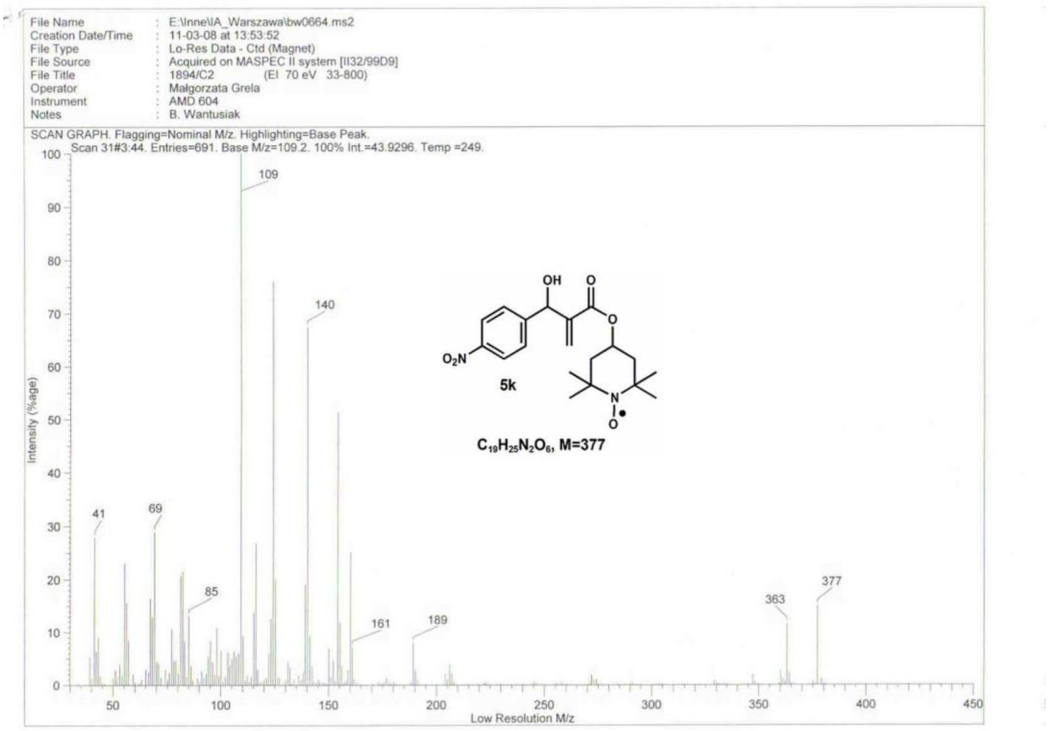

5k, 4-(2-((4-Nitrophenyl)hydroxymethyl)acryloyloxy)-2,2,6,6-tetramethylpiperidine-1-oxyl, R=4-$NO_2C_6H_4$, IR

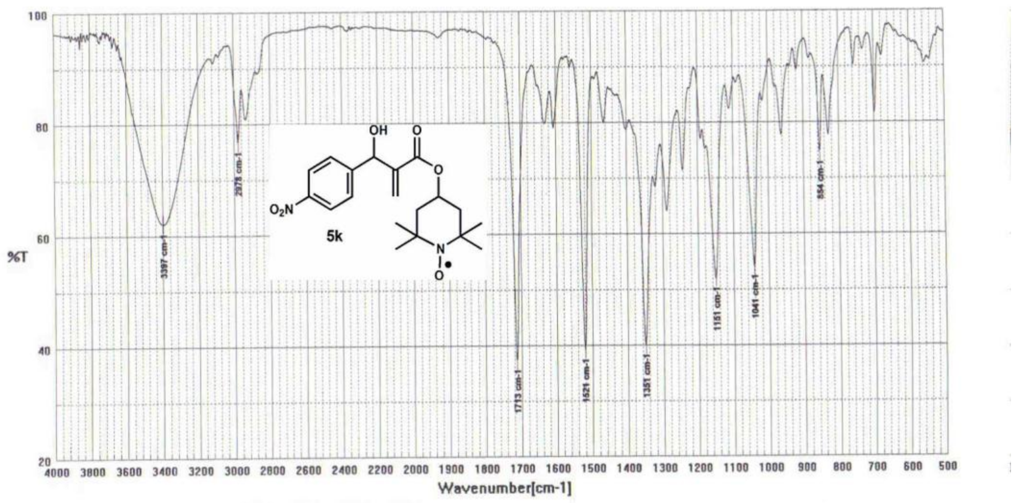

Sample Name 1894/C2 (0,4mg/270mg KBr)
Resolution 1 cm-1
Accumulation 30
Apodization Cosine
Date/Time 110-05-10 9:48

5l, 4-(2-((2,4-Dinitrophenyl)hydroxymethyl)acryloyloxy)-2,2,6,6-tetramethylpiperidine-1-oxyl, R=2,4-di-$NO_2C_6H_3$, EIMS

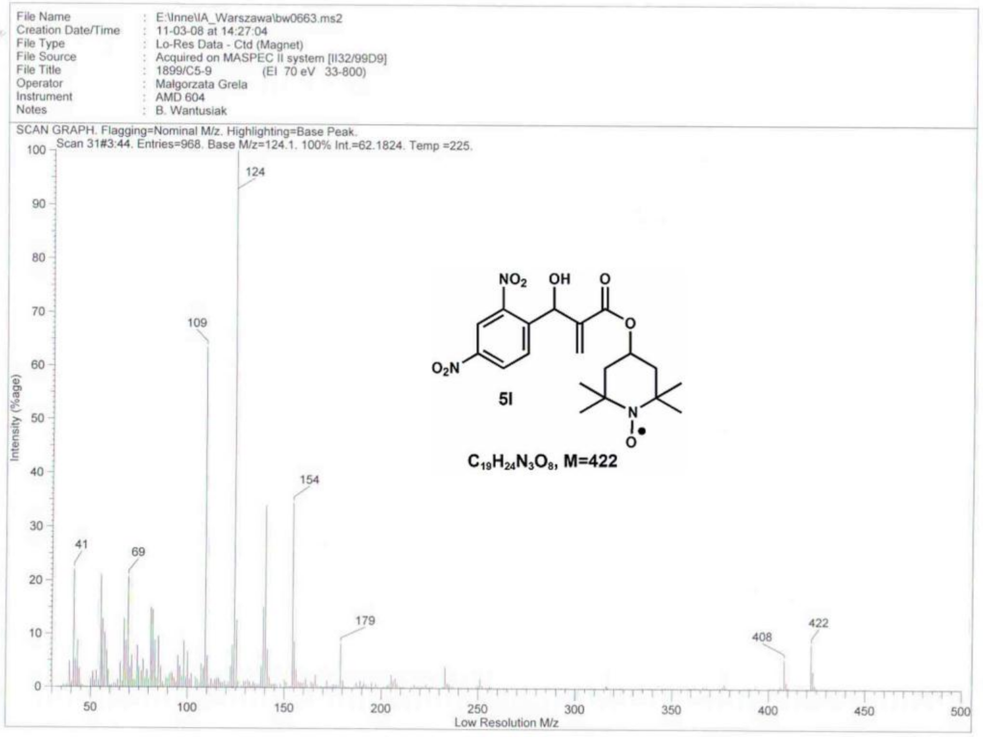

5l, 4-(2-((2,4-Dinitrophenyl)hydroxymethyl)acryloyloxy)-2,2,6,6-tetramethylpiperidine-1-oxyl, R=2,4-di-$NO_2C_6H_3$, IR

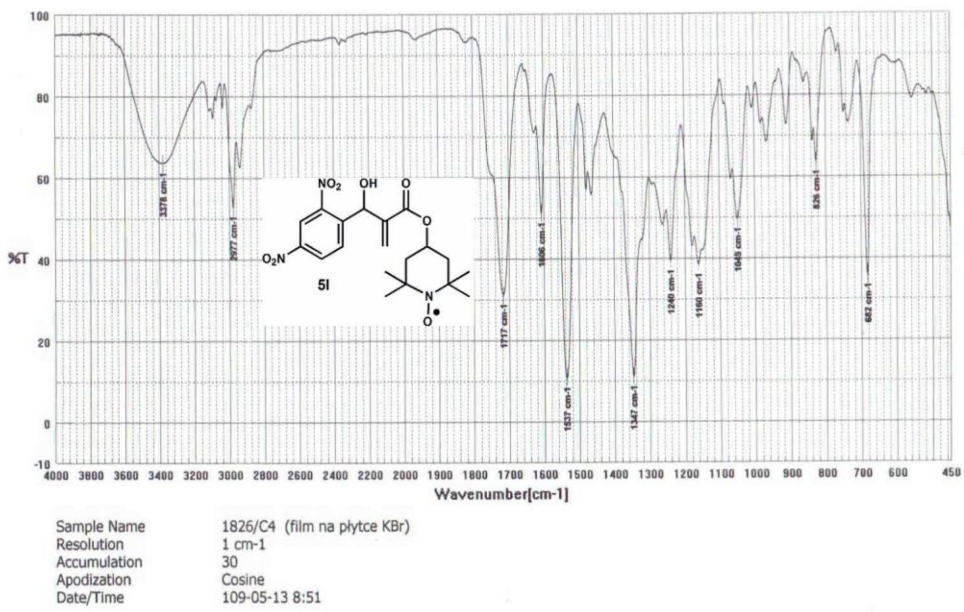

5m, 4-(2-((3-Pyridyl)hydroxymethyl)acryloyloxy)-2,2,6,6-tetramethylpiperidine-1-oxyl, R=3-pyridyl, EIMS

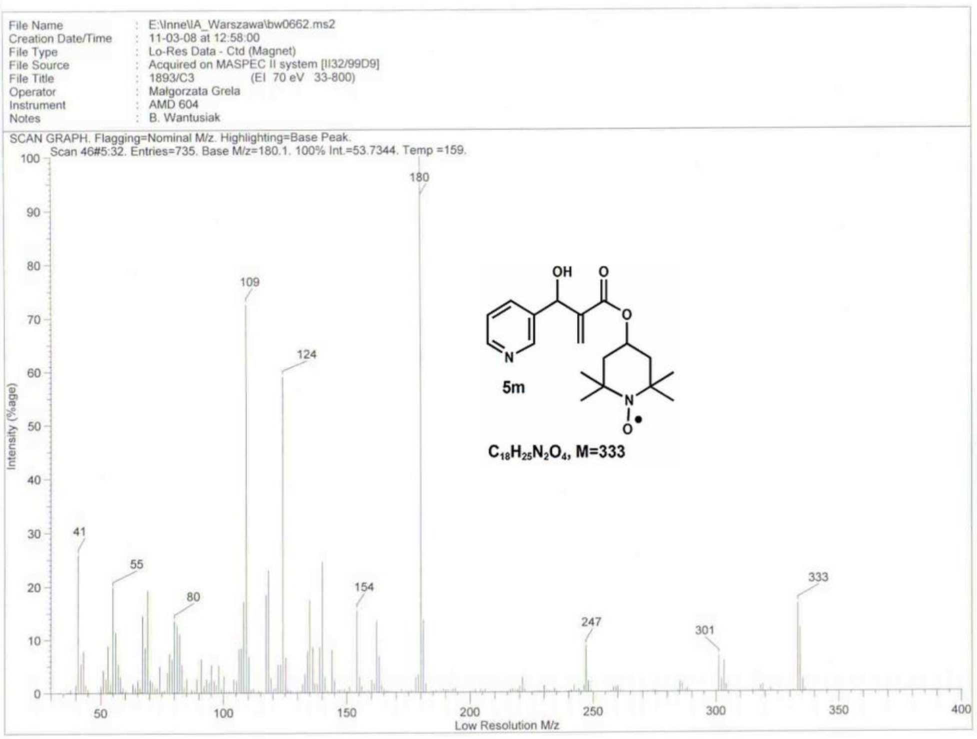

5m, 4-(2-((3-Pyridyl)hydroxymethyl)acryloyloxy)-2,2,6,6-tetramethylpiperidine-1-oxyl, R=3-pyridyl, IR

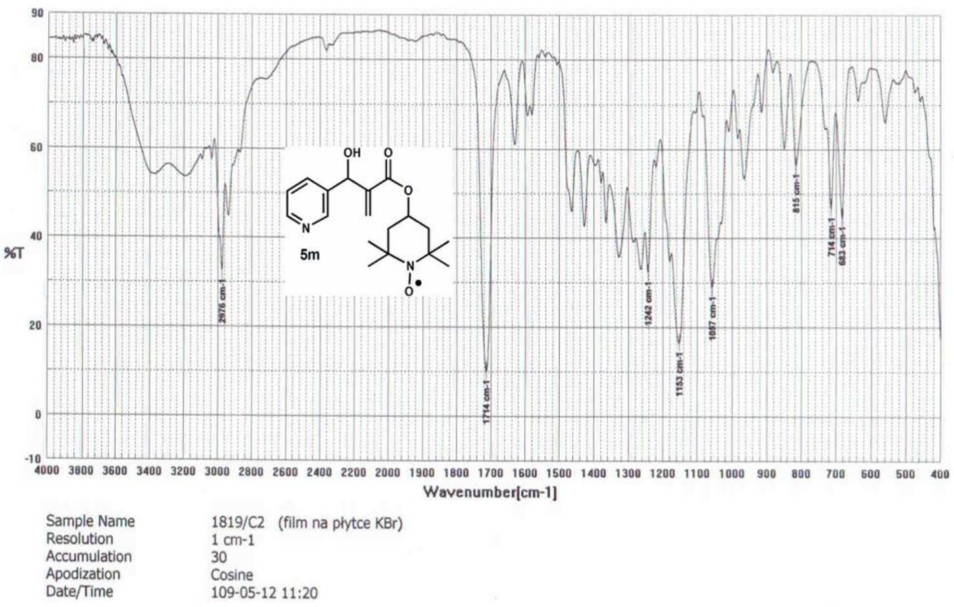

5n, 4-(2-((2-Furyl)hydroxymethyl)acryloyloxy)-2,2,6,6-tetramethylpiperidine-1-oxyl, R=2-furyl, EIMS

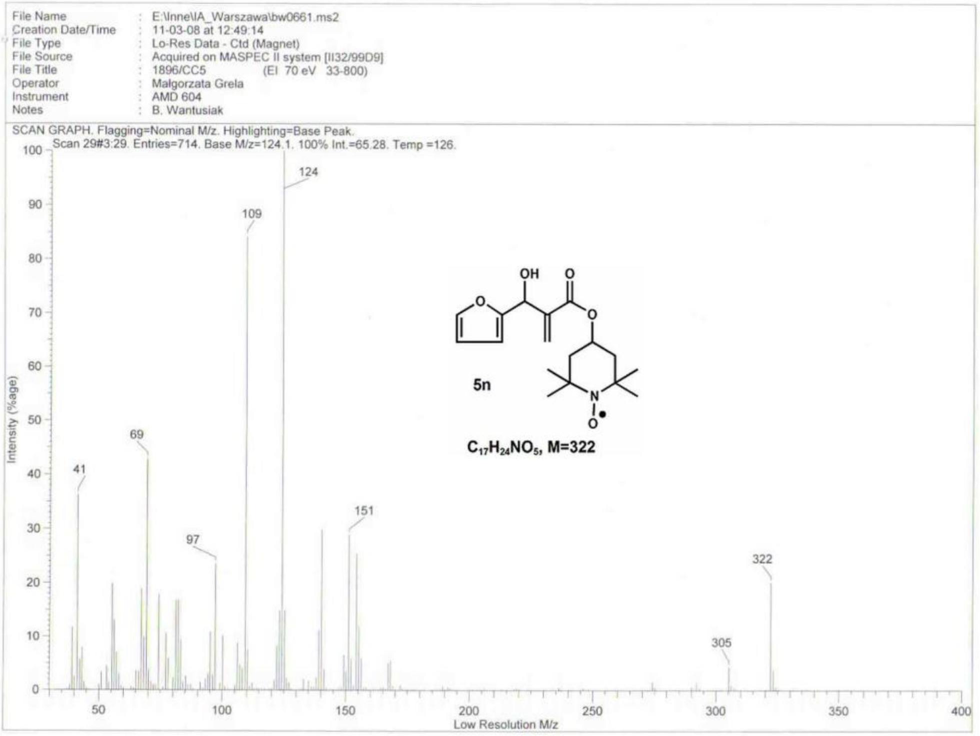

5n, 4-(2-((2-Furyl)hydroxymethyl)acryloyloxy)-2,2,6,6-tetramethylpiperidine-1-oxyl, R=2-furyl, IR

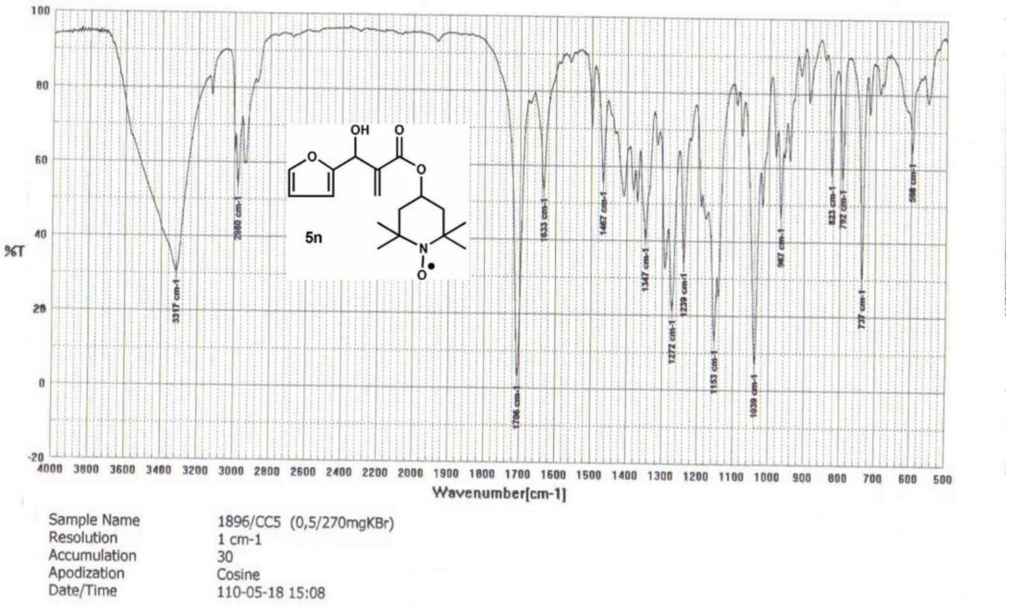

5o, 4-(2-((Ferrocenyl)hydroxymethyl)acryloyloxy)-2,2,6,6-tetramethylpiperidine-1-oxyl, R=ferrocenyl, EIMS

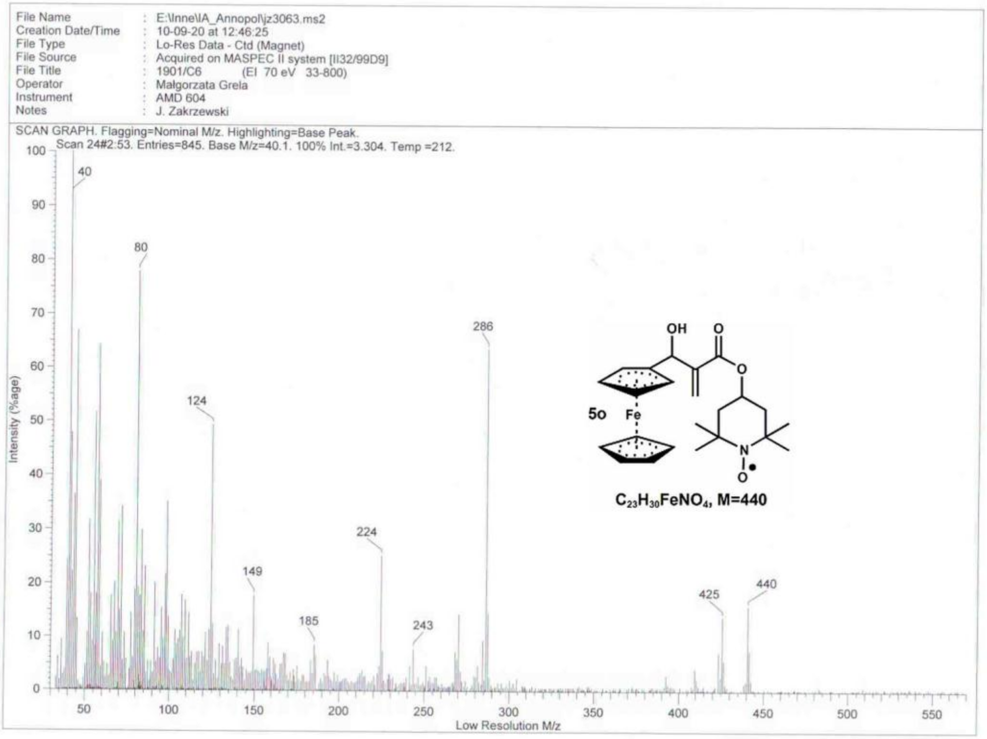

5o, 4-(2-((Ferrocenyl)hydroxymethyl)acryloyloxy)-2,2,6,6-tetramethylpiperidine-1-oxyl, R=ferrocenyl, IR

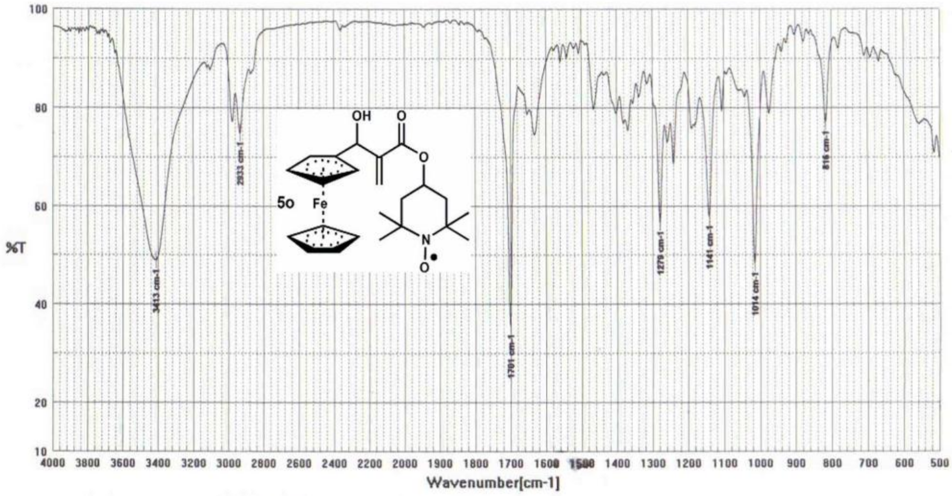

Sample Name	1901/C6 (0,5mg/270mg KBr)
Resolution	1 cm-1
Accumulation	30
Apodization	Cosine
Date/Time	110-09-08 9:51

Blue fractions suggesting the presence of nitroso-compounds during the isolation of **5l**

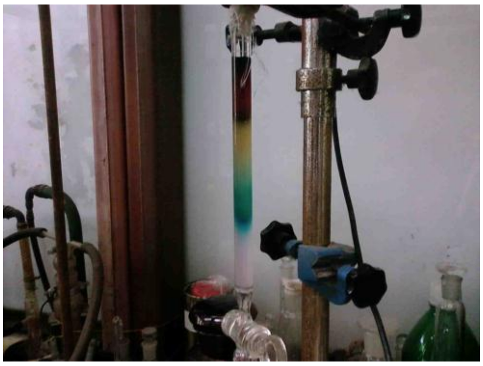

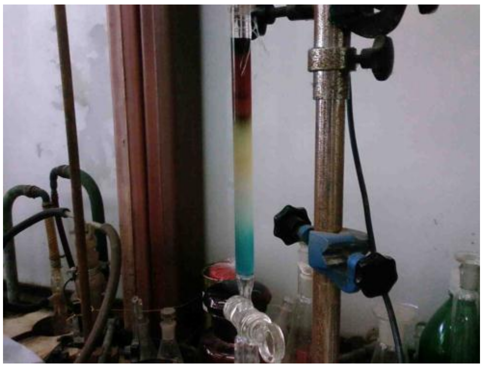

Supplement: File 1 — Detailed spectrographic data. [file Beilstein_J_Org_Chem-08-1515-s001.pdf]
